# Evolution of Multi-Resistance to Vancomycin, Daptomycin, and Linezolid in Methicillin-Resistant *Staphylococcus aureus* Causing Persistent Bacteremia

**DOI:** 10.3389/fmicb.2020.01414

**Published:** 2020-07-07

**Authors:** Chih-Jung Chen, Yhu-Chering Huang, Shian-Sen Shie

**Affiliations:** ^1^Division of Pediatric Infectious Diseases, Department of Pediatrics, Chang Gung Memorial Hospital, Taoyuan City, Taiwan; ^2^School of Medicine, College of Medicine, Chang Gung University, Taoyuan City, Taiwan; ^3^Division of Infectious Diseases, Department of Internal Medicine, Chang Gung Memorial Hospital, Taoyuan City, Taiwan

**Keywords:** methicillin-resistant *Staphylococcus aureus*, persistent bacteremia, vancomycin-intermediate *Staphylococcus aureus*, daptomycin, linezolid, multi-resistance, genomic evolution

## Abstract

The genomic evolution *in vivo* in persistent infection was critical information for understanding how methicillin-resistant *Staphylococcus aureus* (MRSA) was adapted to host environments with high antibiotic selective pressure. Thirty-two successive MRSA blood isolates with incremental non-susceptibility to vancomycin (VISA), daptomycin (DRSA), and/or linezolid (LRSA) were isolated from a patient failing multiple courses of antimicrobial therapy during 1,356 days of bacteremia. Whole genome sequencing (WGS) for all consecutive isolates were conducted to characterize the evolutionary pathways, resistance-associated mutations and their temporal relationship with antimicrobial treatment. The WGS-based phylogeny categorized the isogenic strains into three major clades, I (22 isolates), II (7 isolates), and III (3 isolates), respectively, harboring a median (range) of 7 (1–30), 62 (53–65), and 118 (100–130) non-synonymous mutations when compared to the very first isolate. Clade I strains were further grouped into early and late subclades, which, respectively, shared the most recent common ancestor with Clade III strains at day 393.7 and Clade II strain at day 662.5. Clade I and Clade III strains were characterized, respectively, with high rates of VISA (9/22, 40.9%) and VISA-and-DRSA phenotype (2/3, 66.7%). Linezolid-resistance including VISA-DRSA-and-LRSA phenotype was exclusively identified in Clade II strains after eight courses of linezolid treatment. The LRSA displayed a small colony variant phenotype and were associated with G2576T mutations in domain V region of 23S rRNA. Substantial loss of mobile elements or alleles mediating resistance or virulence were identified during the evolution of multi-resistance. However, the gene loss might not be correlated to the development of VISA, DRSA, or LRSA phenotype. In conclusion, MRSA in persistent bacteremia was adapted to harsh host environment through multiple pathways involving both resistance-associated mutations and extensive gene loss.

## Introduction

Methicillin-resistant *Staphylococcus aureus* (MRSA) bacteremia is a devastating disease associated with high rates of complications and mortality especially in those with underlying conditions (Charles et al., 2004; Tong et al., 2020). Treatment failures including persistent bacteremia were frequently identified in patients with medical implant or prothesis, with greater number of comorbidities and greater disease severity or infections with non-susceptible strains (Charles et al., 2004; Murray et al., 2013; Moise et al., 2016; Yang et al., 2018; Tong et al., 2020). Glycopeptide remains the drug of choice for treatment of MRSA bacteremia though accumulating evidence suggested daptomycin treatment may be associated with improved outcome, especially in patients whose diseases were caused by MRSA strains with reduced susceptibility to vancomycin (Murray et al., 2013; Claeys et al., 2016; Yang et al., 2018). Nevertheless, with the currently available anti-MRSA agents, the clinical and microbiological cure rates of MRSA bacteremia remain unsatisfactory.

Duration of bacteremia was an important factor predicting the outcome of patient with MRSA bloodstream infections (Hamdy et al., 2017). Development of drug resistance during treatment was a common observation in persistent MRSA bacteremia and may play an important role for the failure to eradicate the MRSA strains from bloodstreams (Friedman et al., 2006; Chen et al., 2014b, 2015). An understanding of the *in vivo* evolution of MRSA strains during the development of incremental resistance to the commonly used anti-MRSA agents including vancomycin, daptomycin, and linezolid may help deploy effective therapeutic strategy against the devastating infectious disease.

In the present study, we characterized the genomic evolution in a series of isogenic MRSA blood isolates exhibiting incremental non-susceptibilities to vancomycin, daptomycin, and linezolid. The isolates were belonged to sequence type (ST) 5 clone and consecutively isolated from a patient failing multiple courses of antimicrobial therapy against persistent bacteremia for 3 years before his death. By whole-genome sequencing (WGS), we were able to track all *in vivo* evolutionary clades, determined the phylogenetic relationship between isolates of different clades and identify the genetic alterations including mutations and recombinations associated with resistance to each of the three anti-MRSA agents. We also directly observed a correlation between the use of antimicrobial agents and the genetic changes in distinct evolutionary clades.

## Materials and Methods

### Ethics Statement

The study was approved by the by the institute review boards in Chang Gung Memorial Hospital (CMRPG3C1883), which allowed review of the medical data of the patient. A waiver of consent was granted given the retrospective nature of the project and anonymous analysis of the clinical information of the patient.

### The Case

Approximately 1 month after an aortic repair of aneurysm and replacement with a vascular graft, a middle-aged patient developed MRSA bacteremia on January 10, 2006, for the first time. The bacteremia appeared to be temporarily controlled by administration of vancomycin followed by teicoplanin for 3 months. Unfortunately, the MRSA was cultivated from the bloodstream again in September 08, 2006 (the first available isolate, day 241 of bacteremia) and was afterward identified intermittently in blood cultures in the following 3 years until death on October 20, 2009 (the last available isolate, day 1,356). The anti-MRSA agents including glycopeptides, linezolid, daptomycin, tigecycline, and trimethoprim-sulfamethoxazole were used interchangeably to treat the refractory bacteremia, but the attempts were all unsuccessful. A total of 32 MRSA were isolated from blood cultures, and the isolates gradually developed non-susceptibility to the anti-MRSA agents (Table 1), with the first strain exhibiting vancomycin-intermediate *S. aureus* (VISA) phenotype at day 453 (LTF06, April 08, 2007), the first strain exhibiting both vancomycin-intermediate and daptomycin-resistant (VISA-DRSA) phenotype at day 936 (LTF15, August 03, 2008), and the first strain exhibiting resistance to vancomycin, daptomycin, and linezolid (VISA-DRSA-LRSA) at day 1196 (LTF22, April 20, 2009). Initial genotyping of the 32 strains disclosed that they were belonged to ST5 or its single locus variant, *spa* type t002 or t13754 and universally harboring SCC*mec* II (Supplementary Table S1).

**TABLE 1:** Susceptibilities to 10 non-beta-lactam antimicrobial agents in 32 successive MRSA isolates belonging to CC5-SCC*mec*II and three WGS-defined clades in a patient with persistent bacteremia during 3 years.

Strain	Isolation date (y/m/d)	Number of non-susceptible drug	Antimicrobial agent									
			C	D	E	F	L	S	TE	TG	V	R
Clade I	Median (range)	3 (2–5)										
LTF01	2006/9/8	3	R	S.38	R	S	S 1.5	S	S	S	S 2	I
LTF02	2006/10/27	2	R	S.38	R	S	S 1.5	S	S	S	S 2	S
LTF03	2006/11/20	3	R	S.5	R	S	S 1	S	S	S	S 2	I
LTF04	2007/3/4	3	R	S.5	R	S	S 1.5	S	S	S	S 2	R
LTF05	2007/4/3	3	R	S.5	R	S	S 1.5	S	S	S	S 2	R
LTF06	2007/4/8	4	R	S.75	R	S	S 1	S	S	S	**I 3**	R
LTF07	2007/4/19	3	R	S.75	R	S	S 1	S	S	S	S 2	R
LTF08	2007/7/6	4	R	S.75	R	S	S 1	S	S	S	**I 3**	R
LTF09	2007/7/20	4	R	S.5	R	S	S 1	S	S	S	**I 3**	R
LTF10	2007/8/19	3	R	S.5	R	S	S 1.5	S	S	S	S 2	R
LTF11	2007/11/7	3	R	S.75	R	S	S 2	S	S	S	S 2	R
LTF12	2008/2/13	3	R	S.5	R	S	S 1.5	S	S	S	S 2	R
LTF13	2008/5/7	4	R	S.38	R	S	S 1.5	S	S	S	**I 3**	R
LTF14	2008/6/4	3	R	S.38	R	S	S 1.5	S	S	S	S 2	R
LTF15	2008/8/3	5	R	**R 4**	R	S	S 2	S	S	S	**I 4**	R
LTF19	2009/2/25	3	R	S.75	R	S	S 3	S	S	S	S 2	R
LTF21	2009/4/14	5	R	S.15	R	**I**	S 2	S	S	S	**I 3**	R
LTF27	2009/5/14	3	R	S.75	R	S	S 1.5	S	S	S	S 2	R
LTF28	2009/8/5	4	R	S 1	R	S	S 1.5	S	S	S	**I 3**	R
LTF29	2009/8/11	4	R	S.75	R	S	S 1	S	S	S	**I 3**	R
LTF30	2009/8/16	3	R	S.75	R	S	S 1.5	S	S	S	S 2	R
LTF32	2009/9/27	4	R	S.75	R	S	S 1	S	S	S	**I 3**	R
Clade II	Median (range)	6 (4–7)										
LTF20	2009/3/18	4	R	S.75	R	S	S 3	S	S	S	**I 3**	R
LTF22	2009/4/20	6	R	**R 2**	R	S	**R 8**	S	S	S	**I 3**	R
LTF23	2009/4/24	5	R	S.75	R	**I**	**R 32**	S	S	S	S 2	R
LTF24	2009/4/29	8	R	**R 3**	R	**I**	**R 6**	**R**	S	S	**I 3**	R
LTF25	2009/4/29	7	R	**R 4**	R	**I**	**R 8**	S	S	S	**I 4**	R
LTF26	2009/5/5	6	R	S.75	R	**I**	**R 32**	**R**	S	S	S 2	R
LTF31	2009/9/12	7	R	**R 1.5**	R	S	**R 32**	**R**	S	S	**I 3**	R
Clade III	Median (range)	5 (5–6)										
LTF16	2008/8/16	5	R	**R 6**	R	S	S 1.5	S	S	S	**I 3**	R
LTF17	2008/12/3	5	R	S.75	R	**R**	S.75	**R**	S	S	S 2	R
LTF18	2008/12/12	6	R	**R 4**	R	**R**	S 1	S	S	S	**I 3**	R

*The minimal inhibitory concentrations at mg/L determined by E-test were shown for daptomycin, linezolid, and vancomycin. CC, clonal complex; C, clindamycin; D, daptomycin; E, erythromycin; F, fusidic acid; L, linezolid; R, rifampicin; S, trimethoprim-sulfamethoxazole; TE, teicoplanin; TG, tigecycline; V, vancomycin; WGS, whole genome sequencing. The non-susceptible phenotype (R or I) and the value of minimal inhibitory concentrations to selected antimicrobial agents are highlighted with bold face letters.*

### Strains and Growth Conditions

A total of 32 MRSA strains (LTF01–LTF32, Table 1) were stored at −80°C after their first isolations from the patient. The strains used in this study were grown from their very first stocked cultures. Bacteria was grown in liquid or on solid basic medium (BM) (1% Pepton, 0.5% yeast extract, 0.5% NaCl, 0.1% K_2_HPO_4_, 0.1% glucose) or tryptic soy broth (TSB) (Sigma, Munich, Germany) unless otherwise noted.

To clarify the phylogeny and the evolution rate of the 32 successive MRSA strains, another set of 71 non-duplicated clinical MRSA strains belonging to the same ST5 lineage were obtained from the strain bank. The strains were collected from the major hospitals in Taiwan during 1997 and 2014. The information of the strains including the molecular features had been characterized and presented elsewhere (Huang et al., 2006, 2008; Chen et al., 2010, 2014a).

### SCC*mec* Typing (Detection of *mecA* Gene)

The chromosomal DNA was extracted via QIAamp DNA Mini Kit (Qiagen, CA, United States). All isolates were detected for *mecA* gene and SCC*mec* types by multiplex PCR strategy as described previously (Kondo et al., 2007). The control strains for SCC*mec* types I, II, III, and IVa, kindly provided by Keiichi Hiramatsu, was as follows: type I, NCTC10442; type II, N315; type III, 85/2082; and type IVa, JCSC4744.

### Susceptibility Tests

Susceptibilities to clindamycin, erythromycin, fusidic acid, trimethoprim-sulfamethoxazole, tigecycline, and rifampicin were determined using the standard disk diffusion method according to the CLSI guidelines (CLSI, 2017). Briefly, *S. aureus* isolates were grown in TSB at 37°C to a cell density equivalent to that of a 0.5 McFarland standard. The bacteria were then streaked onto Mueller–Hinton agar plates, and the plates were incubated at 37°C for 24 h before reading. The minimal inhibitory concentrations (MICs) of vancomycin, daptomycin, and linezolid were determined by Etest (bioMe’ rieux) according to the manufacturer’s instructions.

### Colony Morphology

To observe the colony morphologies, the bacteria were refreshed from frozen stock on TSA with 5% sheep blood. A single colony was picked and streaked on BM and TSA with 5% sheep blood, respectively. Agar plates were incubated at 37°C with 5% CO_2_ for 24 h before reading.

### Whole Genome Sequencing (WGS)

The chromosomal DNA was extracted via QIAamp DNA Mini Kit (Qiagen, CA, United States). The WGS of the strains was performed using the next-generation sequencing technology, the Illumina MiSeq sequencer (Illumina, San Diego, CA, United States). The raw data of each strain was cleaned by adapter trimming and exclusion of reads in which greater than 45% of bases were of quality score < 20 (<Q20). A range of 199 million to 318 million bases was generated for each strain, with 71.1- to 113.6-fold base coverage across the genome. This Whole Genome Shotgun project has been deposited at DDBJ/ENA/GenBank under accession number PRJNA495118. The analysis strategy was as follows.

#### *De novo* Assembly of WGS

*De novo* assembly of each of the strains was conducted using SPAdes 3.11.1 (Bankevich et al., 2012). The default *k*-mer lengths of 21, 33, 55, 77, 99, and 127 were set and *–careful* option was used during assembly. The scaffolds outputs greater than 200 bp were used in the following analysis.

#### Phylogenetic Analysis

The multi-alignment of the core genomes of 32 successive MRSA strains was conducted with the parsnp program (Treangen et al., 2014). The ST5 *S. aureus* strain N315 was used as reference strain during the procedure. The multi-alignment file generated by the parsnp script was subsequently used for maximum likelihood phylogeny construction based on the single nucleotide polymorphisms (SNPs) and short insertions or deletions (INDELs) outside of the potential recombination regions with the Gubbins procedure (Croucher et al., 2015). The final phylogenetic tree was input into Interactive Tree of Life^1^ for further annotation.

#### Time-Scaled Phylogeny

In addition to the maximum likelihood phylogeny, the time-scaled phylogeny was constructed with the Bayesian analysis of the molecular sequences using Markov chain Monte Carlo. The BEAST program (v 1.10.4) was used to estimate the clock rates of evolution and the time to the most recent common ancestor (MRCA) (Drummond and Rambaut, 2007). An HKY model with estimated base frequencies, uncorrelated relaxed clock type, with lognormal distribution and constant size of tree prior were used. Three independent chains were run for 100 million generations and sampling every 1,000 generations. The effect sample size values were checked by tracer (v1.7.1) and were greater than 200. A burn-in of 10 million states was removed from each of the three independent runs before combining the results from those runs with the logcombiner program (v 1.10.4) from the BEAST package. The final tree was output and annotated with Figtree program^2^.

#### Annotation of Draft Genome

The annotation was conducted using the Rapid Annotation Server provided by the National Microbial Pathogen Data Resource^3^.

### Identification of VISA- and/or DRSA-Associated Mutations

The cleaned raw data of WGS of 32 consecutive MRSA isolates were, respectively, aligned to the reference genome (N315, GenBank: BA000018.3) using bowtie2 and samtools (Li and Durbin, 2009; Johnson et al., 2014). The variants call was firstly conducted between N315 and 32 MRSA isolates using the bcftools with the command of *call*^4^. The variants call then was conducted between the first isolate (LTF01) and the sequential isolates (LTF02–LTF32) using bcftools with the command of *isec*. The identified variants in LTF02-LTF32 were verified by direct visualization and comparisons of the BAM files of LTF01 and those of the other isolates in the Intergrative Genomics Viewer (Robinson et al., 2011). The variants annotations and predicted effects of SNPs on amino acid alterations were conducted using the snpEFF (Cingolani et al., 2012). The variants appeared in the resistant strains but not in susceptible strains were considered to be associated with drug resistance. The position, effect, and affected alleles of the VISA or DRSA-associated mutations including SNPs and short INDELs were illustrated in the circus plot created with Circa^5^.

### Screening of Interested Genes and Visualization of Recombination

The carriage of plasmids, resistance, and virulence genes among the MRSA strains were detected by srst2 procedure using the PlasmidFinder, ARG-ANNOT and virulence factor of pathogenic bacteria database^6^, respectively. To visualize and compare the draft genomes of 32 stains, the Circular Genome Viewer (CGView) was used to create the map (Stothard and Wishart, 2005).

### Statistics

The descriptive statistics were conducted with Stata 13.0 (StataCorp, College Station, TX, United States). The comparisons of numbers of SNPs or INDELs between vancomycin-susceptible *S. aureus* (VSSA) and VISA strains and numbers of antimicrobial agents to which the strains of different clades were non-susceptible to were conducted with the Mann Whitney non-parametric test method. The comparisons of proportions of non-synonymous SNPs or INDELs and between strains of different genetic clades were conducted with Chi-square or Fisher’s exact test where appropriate. The data was analyzed and figures were output by using the GraphPad Prism 5 for Windows (GraphPad Software, San Diego, CA, United States). *P*-value < 0.05 was considered of statistical significance.

## Results

### WGS-Based Phylogeny Confirmed the Isogenic Nature of Successive Strains

**FIGURE 1:**
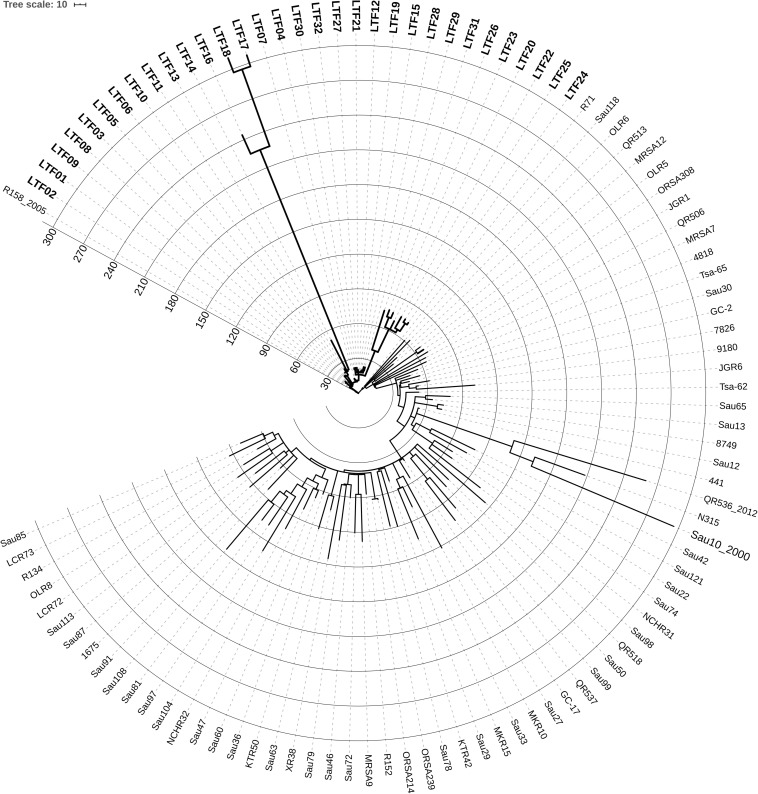
Circular display mode showing the core genome-based phylogeny of 32 successive ST5 MRSA strains (labeled with bold-face letters) from a patient with persistent bacteremia and 71 ST5 MRSA strains (Supplementary Table S2 for detailed information) collected nationwide in Taiwan from 1997 to 2014. The N315 strain belonging to ST5 was included for reference. The numbers at the starts of circles indicates the number of SNPs or short INDELs mutations in the strains, which was larger in certain strains of the persistent bacteremia than in the nationwide strains. The first available MRSA isolate (LTF01) from the patient with persistent bacteremia was closely related to a strain (R158), which was isolated from another patient in the same hospital few years earlier, suggesting the persistent bacteremia was initially caused by the endemic ST5 MRSA in this facility.

The long course of bacteremia across 3 years, the subtle change of ST and *spa* types, and variable antibiograms in the successive strains raised the first question whether all of the successive MRSA strains were derived from the same ancestor or the persistent bacteremia was actually resulted from repeated infections by new strains. To clarify this issue, we conducted the whole genome sequencing of the 32 strains along with a reference set of 71 non-duplicated clonal complex (CC) 5 MRSA clinical isolates collected nationwide from 1997 to 2014 (Supplementary Table S2). The core genome-based phylogeny of the 103 strains disclosed that the 32 successive bacteremic strains were clustered together, which could be clearly distinguished from the other contemporary clinical ST5 isolates (Figure 1). The finding confirmed that the strains causing persistent bacteremia were diverged from the same parental strain and were of isogenic nature.

### WGS-Defined Genetic Clades and Phenotypic Changes of Successive ST5 Strains

**FIGURE 2:**
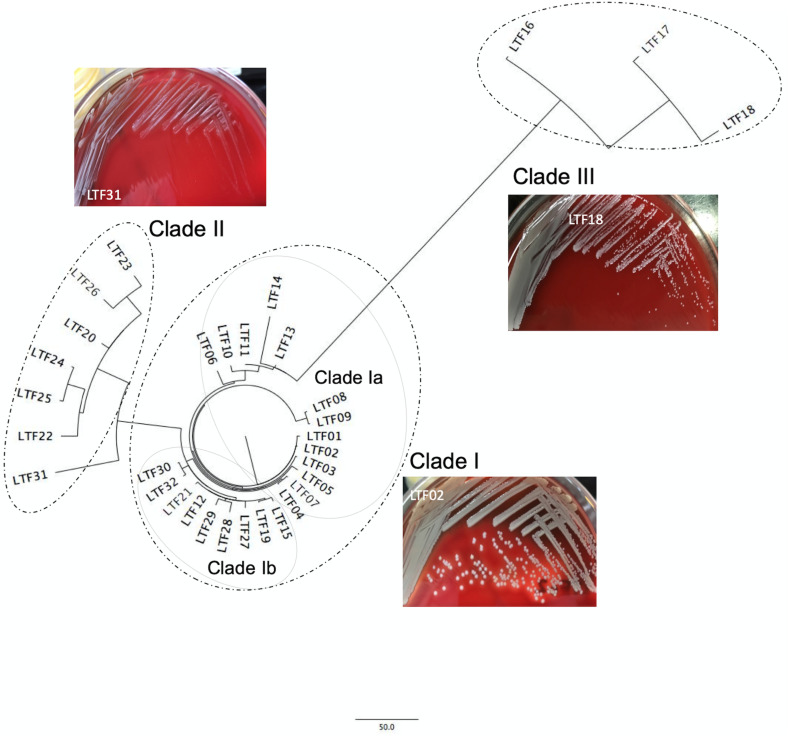
Core genome-based phylogenetic analysis of 32 ST5 MRSA strains from a patient with persistent bacteremia. The successive bacteremic strains were belonged to three major genetic clades with two subclades, Ia and Ib, in Clade I. The bacterial colonies of representative strains on blood agar plates were shown for each clade. The colonies of Clade II strains were at significantly smaller size and whiter color when compared to the strains of Clade I.

When compared to the first isolate (LTF01), a total of 709 SNPs or short INDELs including 320 (45.1%) non-synonymous mutations were identified in the core genomes of the 31 successive isolates (Table 2). The detailed information including the positions of mutations, altered nucleotides, effect of the mutations, and the affected alleles are displayed in the Supplementary Data Sheet S1. Multiple likelihood analysis of the 709 SNP and short INDELs mutations divided the isolates into three major clades (Clades I, II, and III, Figure 2). Clade I strains could be further divided into two subclades, Ia and Ib, respectively, appeared in early course (before day 876 when strain LTF14 was isolated) and late course (after day 764 when strain LTF12 was isolated). The phylogeny demonstrated that strains of Clade III were derived from the early Clade Ia, whereas strains of Clade II were genetically closer to the late Clade Ib.

**TABLE 2:** Numbers of single nucleotide polymorphisms (SNPs) or short insertions/deletions (INDELs) among three clades of MRSA isolates causing persistent bacteremia during 3 years, comparison to the parenteral strain, LTF01.

Isolates	SNPs or short INDELs					
	Total no.	Non-synonymous				
		All	Clade-specific	VISA/DRSA-associated*	*P1*	*P2*
Clade I, 21 strains (%)	136 (100)	77 (56.6)	57 (41.2)	23 (16.9)	<0.001	<0.001
Median (range)	13 (4–69)	7 (1–30)	2 (0–14)	4.5 (1–6)		
Clade II, 7 strains (%)	225 (100)	123 (54.7)	116 (51.6)	51 (22.7)	<0.001	<0.001
Median (range)	106 (91-115)	62 (53–65)	55 (49–60)	13 (5–27)		
Clade III, 3 strains (%)	390 (100)	142 (36.4)	127 (32.6)	12 (3.1)	Comparator	Comparator
Median (range)	341 (287–349)	118 (100–130)	109 (86–115)	6 (3–9)		
All, 31 strains (%)	709 (100)	320 (45.1)	300 (42.3)	86 (12.1)	…	…

*The VISA/DRSA-associated means the non-synonymous SNPs or INDELs were identified in the VISA/DRSA strains but not in the VSSA strains of the same clade. P1 indicates the P-values in the comparison of the proportions of all non-synonymous mutations to total SNPs or short INDELs. P2 indicates the P-values in the comparisons of the proportions of VISA/DRSA-associated non-synonymous mutations to all SNPs or short INDELs.

A significant morphology change of the bacterial colonies was identified for Clade II and Clade III strains. The colonies of the Clade II strains in agar plates were all at tiny sizes and in whiter color compared to the Clade I strains, indicating a small colony variant (SCV) phenotype (Figure 2 and Supplementary Presentation S1). The sizes of the Clade III strains appeared to be in-between of the Clade I and Clade II strains. Along with the morphological change, the Clade II strains also exhibited greater drug resistance when compared to the strains in Clade I. A range of 4–7 (median, 6) non-susceptible antimicrobial agents was identified in Clade II strains, which was significantly greater than the numbers of non-susceptible drugs in Clade I strains (2–5 drugs [median, 3], *P* < 0.001, Table 1). The LRSA strains including those of VISA-DRSA-LRSA phenotype were identified exclusively in strains of Clade II (LTF22, 24, 25, and 31).

### Mutations and Evolution Rates of ST5 MRSA Strains in Persistent Bacteremia Versus Those in General Health-Care Facilities

The proportions of non-synonymous mutations were similar in strains of Clade I and Clade II (56.6% vs. 54.7%, *P* = 0.718, Table 2) but was significantly lower in strains of Clade III (36.4%, *P* < 0.001 for both comparisons to Clade I and Clade II, Table 2). A majority (300, 93.8%) of the 320 non-synonymous mutations occurred in only strains of certain clade and were considered clade-specific. The remaining 20 mutations harbored by strains of different clades are listed in Supplementary Table S3. The distributions of the 20 mutations among the strains further supported that the Clade II and Clade Ib strain shared the MRCA, whereas Clade III strains were more likely derived from Clade Ia.

To estimate the rate of evolution in the bacteremic strains of persistent infections, the BEAST program was applied using the reference set of 71 CC5 isolates (Supplementary Table S2) as the comparator. The mean clock rate for the 32 successive strains was 1.4518E-5 [95% highest posterior density (HPD) interval, 8.6295E-6, 2.0502E-5], which was approximately 6.4-fold faster than the clock rate of the nationwide collection isolates (mean clock rate, 2.2845E-6, 95% HPD interval, 2.0256E-6, 2.5491E-6). Further, the accumulation number of SNPs or short INDELs reached >300 in some of the successive bacteremic strains within 3 years, whereas a majority of the nationwide isolates in the reference set had less than 180 mutations during a time frame of 17 years (1997–2014) (Figure 1).

### Recombinations in the Successive Strains

**FIGURE 3:**
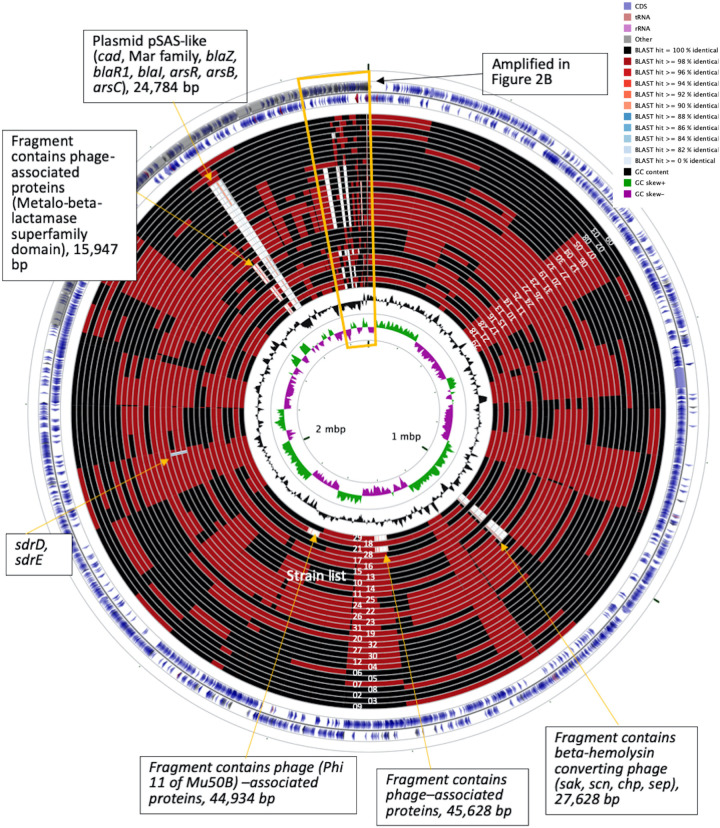
CGview of the genome map of 32 MRSA isolates from a patient with persistent bacteremia. The empty regions indicated missing of the plasmids or chromosomal fragments.

**FIGURE 4:**
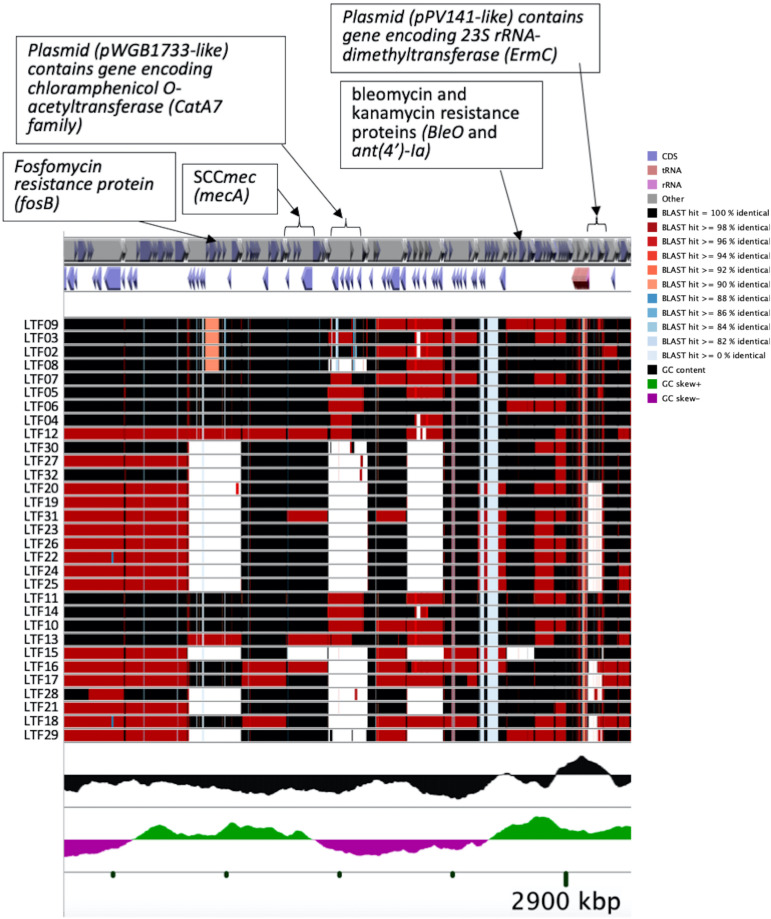
CGview of the genome map of 32 MRSA isolates from a patient with persistent bacteremia. Amplification of a region containing two plasmids (pWGB1733-like and pPV141-like) and drug-resistant genes in Figure 3.

**FIGURE 5:**
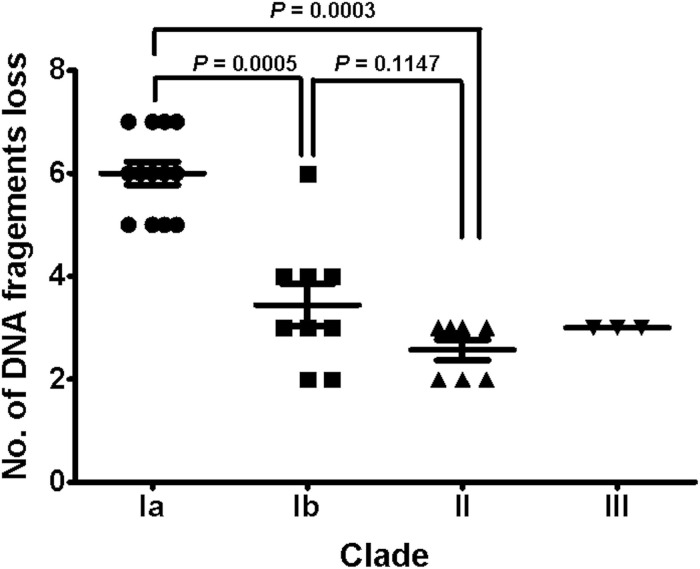
The distributions of numbers of gene loss in different clades of 32 successive MRSA isolates. The numbers of gene loss were significantly greater for strains (Clades Ib and II) isolated in later course of bacteremia than for strains (Clade Ia) in early course. Comparison to isolates in Clade III was not done due to the small number of isolates.

To understand the role of recombinations during the evolution of MRSA strains exhibiting increasing drugs resistance in this case, we compared 32 strains by screening the plasmid, resistance, and virulence genes with the srst2 procedure and viewing the graphic genome maps with the CGview program (Table 3 and Figures 3, 4). The srst2 procedure detected three plasmids, which were structurally similar to pSAS (BX571858), pWGB1773 (EF537646), and pPV141 (U82607) in the first three strains (LTF01, LTF02, and LTF03). The plasmids were gradually lost in the successive strains and complete missing in two strains of Clade Ib (LTF19 and LTF28) and all strains of Clade II. Several prophage-associated fragments were also missing in some of the successive strains. Of them, the beta-hemolysin converting prophage containing the immune evasion cluster (*sak*, *scn*, *chp*, and *sep*) was lost in some Clade I strains and all three strains of Clade III. The other lost resistance/virulence genes including *fosB*, *mecA*, *bleO*, *ant*(*4*′)*-Ia*, *sdrD*, and *sdrE* appeared not to be prophage or plasmid related. Together, a total of 11 recombination patterns involving three plasmids and four resistance/virulence genes-contained fragments were identified among the 32 successive strains (Table 3). The numbers of gene loss were significantly greater in the strains isolated in the later course of bacteremia (Clade Ib and Clade II strains, *P* = 0.0005 and *P* = 0.0003, respectively) compared to the Clade Ia strains being isolated at early course of bacteremia (Figure 5).

**TABLE 3:** Distributions of mobile elements and alleles encoding molecules of drug resistance or virulence in 32 successive MRSA strains isolated from a patient with persistent bacteremia in 3 years.

Clade	Strain	Resistant phenotype to VAN, DAP, and LZD	Mobile elements or alleles of resistance/virulence							Recombination pattern
			pSAS	pWGB1733 (*catA7*)	f*osB*	pPV141 (*ermC*)	*mecA, bleO, ant(4′)-Ia*	IEC (*sak, scn, chp, sep*)	*sdrD sdrE*	
Ia	LTF01	…	+	+	+	+	+	+	+	A
Ia	LTF09	VISA	+	+	+	+	+	+	+	A
Ia	LTF03	…	+	+	+	+	+	+	+	A
Ia	LTF02	…	+	+	+	+	+	+	+	A
Ia	LTF08	VISA	+	−	+	+	+	+	+	B
Ia	LTF07	…	−	+	+	+	+	+	+	C
Ia	LTF05	…	−	+	+	+	+	+	+	C
Ia	LTF06	VISA	−	+	+	+	+	+	+	C
Ia	LTF04	…	−	+	+	+	+	+	+	C
Ib	LTF12	…	−	+	+	+	+	+	+	C
Ib	LTF30	…	−	−	−	+	+	+	+	D
Ib	LTF27	…	−	−	−	+	+	+	+	D
Ib	LTF32	VISA	−	−	−	+	+	+	+	D
II	LTF20	VISA	−	−	−	−	+	+	+	E
Ib	LTF19	…	−	−	−	−	+	+	+	E
II	LTF31	VI-DR-LRSA	−	−	−	−	+	+	+	E
II	LTF23	LRSA	−	−	−	−	+	+	+	E
II	LTF26	LRSA	−	−	−	−	+	+	+	E
II	LTF22	VI-DR-LRSA	−	−	−	−	+	+	−	F
II	LTF24	VI-DR-LRSA	−	−	−	−	+	+	−	F
II	LTF25	VI-DR-LRSA	−	−	−	−	+	+	−	F
Ia	LTF11	…	−	+	+	+	+	−	+	G
Ia	LTF14	…	−	+	+	+	+	−	+	G
Ia	LTF10	…	−	+	+	+	+	−	+	G
Ia	LTF13	VISA	−	+	+	+	+	−	+	G
Ib	LTF15	VI-DRSA	−	−	−	+	−	+	+	H
III	LTF16	VI-DRSA	−	−	+	*	+	−	+	I
III	LTF17	…	−	−	+	−	+	−	+	I
Ib	LTF28	VISA	−	−	−	−	+	−	+	J
Ib	LTF21	VISA	−	−	−	+	+	+	+	K
III	LTF18	VI-DRSA	−	−	+	*	+	−	+	I
Ib	LTF29	VISA	−	−	−	−	+	−	+	J

*The drug-resistant genes carried in the plasmids included cadD mediating cadmium resistance, blaz, blaR1, and blaI mediating penicillin resistance in the pSAS-like plasmid, catA7 encoding chloramphenicol O-acetyltransferase mediating chloramphenicol resistance in the pWGB1733-like plasmid and ermC encoding 23S rRNA-dimethyltransferase mediating erythromycin resistance in a pPV141-like plasmid. The strains were listed in accordance with the positions in Figure 3. GenBank Accession: pSAS, BX571858; pWGB1773, EF537646; pPV141, U82607; pUB110, X03408.1. ‘+’ and shadowed cells indicates presence of the mobile elements or alleles, ‘−’ and empty cells indicates absence of the mobile elements or alleles, ‘^∗^’ indicates presence of emrC but absent for the pPV141 plasmid.*

### Correlations of Genetic Alterations and Development of Multi-Drug Resistance

**FIGURE 6:**
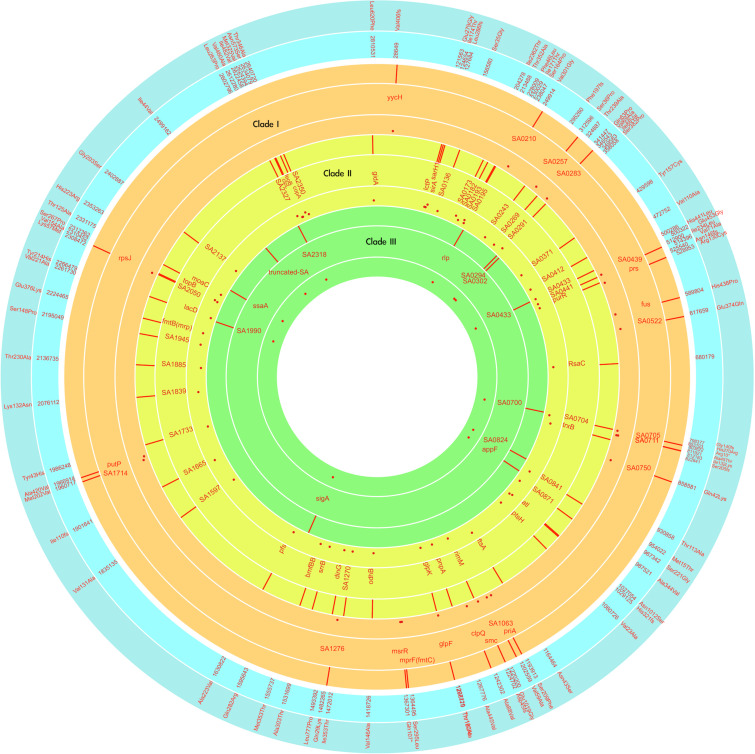
Map of the VISA/DRSA-associated genetic alteration among the successive MRSA strains of three clades marked with different colors, with the outer ring showing the position, middle ring showing the affected alleles, and inner ring showing the rates of affected strains in the indicated clade. Inner and outer ring in blue color, respectively, indicates the positions (in N315) and effects of mutations. The red dot indicates the carriage rate of the indicated mutation for strains in that clade. The plot was generated with the Circa program.

The VISA phenotype (vancomycin MIC of 3–4 mg/L) occurred in strains at an intermittent manner since the isolation of LTF06 and accounted for 40.9, 71.4, and 66.7% of strains of Clades I, II, and III, respectively. The DRSA phenotype was exclusively identified in strains with VISA phenotype irrespective of clades, suggesting a linked machinery of incremental non-susceptibility to vancomycin and daptomycin. A total of 87 mutations in 86 alleles occurring in VISA or VISA-DRSA strains but not in VSSA strains were considered to be associated with VISA/DRSA phenotypes and are displayed in Figure 6 and Supplementary Data Sheet S2. Of them, nine mutations in eight alleles associated with VISA/DRSA phenotype that had been reported in the literature are listed in Table 4. One isolate (LTF15) exhibited resistance to daptomycin among Clade I strains harbored a gain-in-function mutation in *mprF* (S295L) (Figure 6), which was not identified in the other VISA-DRSA strains in Clades II and III. When compared to the VSSA strains, the VISA or VISA-DRSA strains tended to harbor greater numbers of non-synonymous and clade-specific mutations (Supplementary Table S4).

**TABLE 4:** Selected genetic alterations associated with vancomycin-intermediate *S. aureus* (VISA) and/or daptomycin-resistant *S. aureus*-associated (DRSA) phenotypes in successive ST5 MRSA strains causing persistent bacteremia.

Nucleotide position in N315 genome	Reference nucleotide(s)	Altered nucleotide(s)	Affected allele	Amino acid change	Function of affected allele^#^	Gene function class^#^	Reference(s) of reported association with VISA/DRSA phenotype
28649	G	GA	*yycH*	Val406fs	YycH domain signal transduction protein, putative	Protein fate	Mwangi et al., 2007; Gardete et al., 2012; Chen et al., 2015; Cameron et al., 2016
528953	C	T	*prs*	Arg130Cys	Ribose-phosphate pyrophosphokinase	Purines, pyrimidines, nucleosides, and nucleotides	Mwangi et al., 2007; Song et al., 2013; Yamaguchi et al., 2019
1364495	C	T	*mprF* (*fmtC*)	Ser295Leu	Phosphatidylglycerol lysyltransferase	Unknown function	Yang et al., 2009; Rubio et al., 2010; Chen et al., 2015
1367301	C	T	*msrR*	Gln107*	Regulatory protein MsrR	Regulatory functions	Kato et al., 2009; Katayama et al., 2016
525540	CA	C	*purR*	Asn146fs	Pur operon repressor	Purines, pyrimidines, nucleosides, and nucleotides	Fox et al., 2007; Renzoni et al., 2017; Miller et al., 2020
1027054	T	C	*atl*	Asn1012Ser	Bifunctional autolysin	Transport and binding proteins	Wootton et al., 2005; Utaida et al., 2006; John et al., 2011; Cafiso et al., 2012, 2014
1029125	CGT	C	*atl*	His321fs	Bifunctional autolysin	Transport and binding proteins	Wootton et al., 2005; Utaida et al., 2006; John et al., 2011; Cafiso et al., 2012, 2014
1267776	C	T	*pnpA*	Ala445Val	Polyribonucleotide nucleotidyltransferase	Transcription	Song et al., 2013
2353263	A	G	*ssaA*	His223Arg	CHAP domain-containing protein	Hypothetical protein	McAleese et al., 2006; McEvoy et al., 2013; Cameron et al., 2016

*Only the genes with reported association with VISA/DRSA phenotype are listed. ^#^The function and class of affected genes were abstracted from AureoWiki website (Fuchs et al., 2018).*

Resistance to rifampin was firstly identified in strains LTF04, which harbored a non-synonymous SNP (L527L) in *rpoB* gene. The other rifampin-resistant strains also had mutations in *rpoB* gene though with distinct SNPs (i.e., L482A, S529L, S717L, Supplementary Data Sheet S1). Another VISA strain (LTF21) exhibited resistance to fusidic acid, which was associated with the H438P mutation in *fus* gene (Figure 6). The resistance to linezolid was associated with mutations in 23s rRNA. All of the linezolid-resistant strains harbored two to three G2576T mutations in domain V regions of 23S rRNA (Table 5).

**TABLE 5:** Frequency of G2576T mutations in domain V regions of isolates with resistance to linezolid.

Strain.	Linezolid, MIC (mg/L)	23S position				
		P1-2576	P2-2576	P3-2576	P4-2576	P5-2576
LTF01	S, 1.5	G	G	G	G	G
LTF22	R, 8	G	G	T	G	T
LTF23	R, 32	G	G	T	G	T
LTF24	R, 6	G	G	T	G	T
LTF25	R, 8	G	G	T	G	T
LTF26	R, 32	G	G	T	G	T
LTF31	R, 32	G	G	T	T	T

*A parental strain (LTF01) with susceptibility to linezolid was used as control. MIC, minimal inhibitory concentration at mg/L.*

### Time-Scaled Phylogeny and Antimicrobial Treatment Course

**FIGURE 7:**
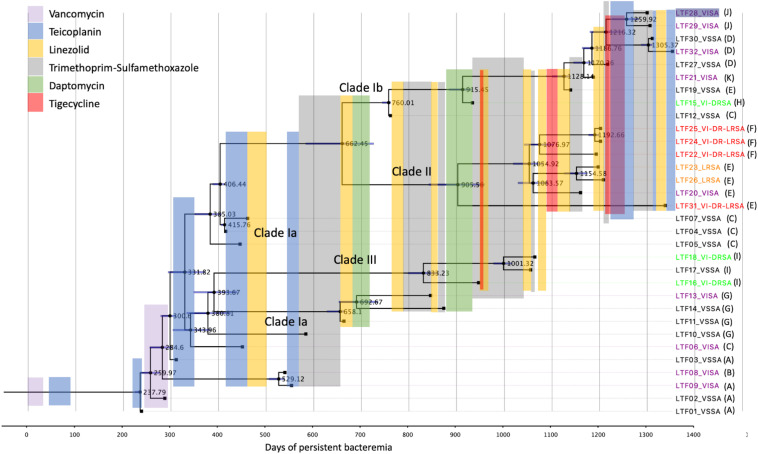
Antimicrobial treatment courses and the time-scaled phylogeny of 32 successive MRSA strains in a patient with persistent bacteremia by BEAST program. The node ages are shown as days after the onset of bacteremia, and the 95% highest poster density are displayed as blue bars. The tips are labeled with strain names followed by drug-resistant phenotypes and the patterns of recombinations indicated in Table 3. VISA, vancomycin-intermediate *S. aureus*; VSSA, vancomycin-susceptible *S. aureus*; VI-DRSA, vancomycin-intermediate and daptomycin-resistant *S. aureus*; LRSA, linezolid-resistant *S. aureus*; VI-DR-LRSA, vancomycin-intermediate, daptomycin-resistant, and linezolid-resistant *S. aureus.*

The time-scaled phylogeny of the 32 bacteremic strains and the use of antimicrobial agents in different time frames during the persistent bacteremia are shown in Figure 7. For most of the time, the patient received monotherapy for the bacteremia, with glycopeptide being mainly used between day 0 and 600. A majority (10 of 13 strains) of the Clade Ia strains were isolated in this period, with strains LTF11, LTF13, and LTF14 being the exceptions that shared the MRCA (day 393.7) with Clade III strains and survived beyond day 600. The MRCA of strains of Clade Ia (LTF04, LTF05, and LTF07), Clade Ib, and Clade II were also predicted to appear during this time frame at day 385.0.

Between days 600 and 1,200, monotherapy with TMP-SXT, linezolid, daptomycin, and tigecycline was interchangeably used. Combination therapy with tigecycline followed by linezolid was only shortly used with TMP-SXT to treat the VISA-DRSA strains (LTF15 and LTF16) in this period. The use of a second course of daptomycin successfully eradicated the remaining Clade Ia strains LTF11, LTF13, and LTF14 but rapidly select the VISA-DRSA strains of Clade III (LTF16 and LTF18) and Clade Ib (LTF15). The VISA-DRSA-LRSA strains of Clade II started to emerge at the end of this time frame. After day 1,200, because of the continually isolated VISA-DRSA-LRSA strains, combination therapy with tigecycline and glycopeptide was launched. Unfortunately, the attempt was unsuccessful and one Clade II strain of VISA-DRSA-LRSA phenotype (LTF31) remained being isolated. In addition, the regimen was unable to stop the less-resistant strains, and three VISA strains and one VSSA strain of Clade Ib survived the intensive combination treatment.

## Discussion

Our study demonstrated that to adapt to the host environment with high antibiotic selective pressure, the MRSA ST5 isolates underwent rapid genetic evolution through three major pathways from strains of Clade Ia, respectively, to Clade Ib, Clade II, and Clade III. The genetic alterations during the evolution involved both mutations (SNP or short INDELs) and substantial genes loss likely by recombinations. It has been well documented that mutations in certain alleles in MRSA strains could lead to increased non-susceptibility to the anti-MRSA agents of last resort, including vancomycin, daptomycin, and linezolid (Howden et al., 2010; Gu et al., 2013; Yang et al., 2013; Chen et al., 2015). By comparing the mutations among VISA/DRSA strains to the VSSA strain in the same clade, we were able to identify dozens of mutations potentially associated with VISA/DRSA phenotypes (Figure 6 and Supplementary Data Sheet S2). Indeed, several alleles harboring mutations (i.e., *rpoB*, *mrpF*, and 23S rRNA) identified in the current study had been previously confirmed to be accountable for the VISA, DRSA, or LRSA phenotypes (Matsuo et al., 2011; Gu et al., 2013; Yang et al., 2013). Of note, the G2576T mutation in domain V regions of 23S rRNA has been shown to be one of the major machineries of linezolid resistance and was exclusively identified in all isolates resistant to linezolid in this study. Further, the only isolate (LTF15) exhibiting resistance to daptomycin in Clade I harbored a point mutation S295L in *mprF* gene. MprF is the synthase for positively charged lysylphosphatidyl-glycerol (L-PG), which is considered to be one of the major contributors to the positive charge of *S. aureus* bacterial surface. The MprF_S__295__L_ has been demonstrated to be a gain-in-function mutation associated with enhancing the synthesis of L-PG and reducing the binding of daptomycin to *S. aureus* (Yang et al., 2013). Moreover, correlation of resistance to other anti-MRSA agents including rifampin and fusidic acid and the mutations in *rpoB* (Lys482Asn, Ile527Leu, Ser529Leu, and Ser717Leu) and *fus* (Val86Ala) genes, respectively, was also identified in this study (Table 1 and Supplementary Data Sheet S1). The data combined together strongly suggested the SNPs/INDELs mutations played an important role in MRSA exhibiting incremental drug resistance. The importance of mutations in resistance to vancomycin or daptomycin was further supported by the finding that the VISA/DRSA strains harboring a significantly greater number of SNPs and/or INDELs compared to the VSSA strains (Supplementary Table S4).

In addition to *mprF*, at least seven alleles with mutations identified in the current study had ever been reported to be associated with VISA/DRSA phenotypes (Table 4). Of them, the alleles including *yycH*, *atl*, and *ssaA* are involved in the *WalKR* cell wall regulatory operon controlling cell wall synthesis and autolysis (Dubrac et al., 2008). Of note, the frameshift mutation of *yycH* resulting in truncated YycH in VISA/DRSA strains was reported in previous studies by us and other groups (Mwangi et al., 2007; Gardete et al., 2012; Chen et al., 2015; Cameron et al., 2016). *yycH* together with *yycI* and *yycJ* is located directly downstream of the *walKR* two-component regulatory (TCR) system and belonged to the *walKR/yycHIJ* operon. It has been demonstrated that the YycH and YycI interact with the sensor kinase WalK and had positive regulatory role for *walKR* TCR (Szurmant et al., 2007). Mutations in *yycH* or *yycI* in VISA strains had a deleterious impact on the YycHI/WalKR complex through down-regulation of *walKR* regulon resulting in impaired autolysis. Allele *atl* together with another gene *lytM* are autolysins genes encoding the products majorly involved in the cell-wall expansion, remodeling, and daughter cell separation under the regulation of the *WalKR* system (Dubrac et al., 2008). Mutations or down-regulation of *atl* had been shown to be associated with VISA phenotype most likely by reducing autolysis and increased thickness of the cell wall (Howden et al., 2010). *ssaA* is also an autolysin gene encoding protein with CHAP amidase domain and regulated by WalKR system through directly binding by WalR to the promoter region (Dubrac et al., 2008; Howden et al., 2011). It has been demonstrated that the expression of s*saA* was down-regulated in strains exhibiting VISA phenotype (McAleese et al., 2006; Cameron et al., 2016).

The VISA/DRSA-associated alleles including *prs* and *purR* were belonged to functional class of purines, pyrimidines, nucleosides, and nucleotides. Association of *prs* mutation and VISA or DRSA phenotype had been reported in at least three studies, but the role of this gene on drug resistance had not been directly addressed (Mwangi et al., 2007; Song et al., 2013; Yamaguchi et al., 2019). The *prs* gene encodes ribose-phosphate pyrophosphokinase, and mutation in *prs* may have a negative effect on the biosynthesis of purine and pyrimidine and further affect the growth rate of bacteria. However, the importance of *prs* mutation on drug resistance requires further study and should be carefully evaluated. Indeed, as shown in previous reports, mutation in another putative purine regulator, *purR*, has been observed in VISA/DRSA strains but a casual association was excluded by follow-up study investigating its biochemical and physiological characteristics (Fox et al., 2007). The other two VISA/DRSA-associated alleles (*msrR* and *pnpA*) are of regulatory or transcriptional function. Introduction of a mutation (E146K) in *msrR* had been reported to convert the VSSA strain of N315 background into heterogenous VISA phenotype (Katayama et al., 2016). *msrR* is a member of the LytR-CspA-Psr family of cell envelope-related transcriptional attenuators and was shown to be inducible by cell wall active agents, such as β-lactams, glycopeptides, and lysostaphin (Kato et al., 2009). It was suggested that the *msrR* mutation promotes the tethering of wall teichoic acid and the capsule to the cell wall, which then leads to decreased autolytic activity and the development of VISA phenotype. Allele *pnpA* is a gene-encoding polyribonucleotide nucleotidyltransferase, and mutation of this gene had ever been identified in a laboratory-derived DRSA strain (Song et al., 2013). PnpA is involved in the quality control of RNA precursors in *Escherichia coli* and appeared to have a role in the persistent infection of *Salmonella enterica* (Clements et al., 2002; De Lay and Gottesman, 2011). Its role in the resistance to vancomycin and/or daptomycin in *S. aureus* remained not determined.

In contrary to the SNPs or INDELs mutations, the role of recombination during the incremental drug resistance in persistent MRSA infections was largely unknown. In the current study, recombinations with the forms of loss of plasmids, loss of prophage with or without carrying virulence/resistance genes, and missing certain genetic fragments unrelated to plasmids or prophage (i.e., *sdrD*, *sdrE*, and *fosB*, Table 3) appeared to be common events during the evolution. It was intriguing to learn that the numbers of gene losses were greater in the strains being isolated at the later course of bacteremia (Clade Ib, Clade II, and Clade III strains) but appeared not necessarily related to the development of drug resistance. Of note, the susceptible strain (e.g., LTF19, VSSA strain in Clade Ib) had the same pattern of recombination as the strains in Clade II with multi-resistance to vancomycin, daptomycin, and linezolid (LTF31, VI-DR-LRSA, Table 3 and Figure 7). The observation suggested that the recombination leading to reduction in genome size may not be the critical step for the development of drug resistances but may be an important advantage for the fitness and persistence of MRSA strains in the extremely harsh condition (Albalat and Cañestro, 2016). Another intriguing observation was the loss of a fragment in SCC*mec* containing *mecA* and other two drug-resistant genes [*bleO* and *ant*(*4*′)*-Ia*] in the strain LTF15 exhibiting resistance to vancomycin and daptomycin. The strain was also of high-level resistance to beta-lactams including oxacillin and cefoxitin with minimal inhibitory concentrations of 32 mg/L and >256 mg/L, respectively. The *mecA*-negative MRSA has been reported elsewhere and the proposed machinery mediating beta-lactam resistance included mutation in other genes encoding penicillin-binding proteins (PBP) (i.e., PBP4) and through regulatory pathways (Banerjee et al., 2010; Roemer et al., 2013). A follow-up study is ongoing to address the mechanism of beta-lactams resistance in the MRSA strain absent for *mecA* gene.

The aggressive antimicrobial treatment in this patient was believed to be the major driving force of the evolution, which fostered a 6.4-fold faster clock rate of mutations in this particular case than that in other clinical isolates in the general health-care settings. The molecular evolution rate is generally determined by a range of factors including the background mutation rate, the strength of selection, generation time, and population size (Albalat and Cañestro, 2016). In this study, the “strength of selection” was majorly coming from aggressive antimicrobial therapy. To minimize the confounding effects of background mutation rate, generation time, and population size, we selected a group of MRSA strains with the same genetic background (CC 5) as the comparator. The comparator strains were non-duplicated clinical isolates collected from different patients nationwide during a time span of 17 years. They were not accountable for persistent infections or from longitudinal studies and should be suitable for estimating the evolution rate of contemporary MRSA in the healthcare facilities.

The initial glycopeptide treatment courses in this case was unsuccessful, which was not only failure to completely eradicate the Clade Ia strains but rapidly select a couple of VISA strains of the same clade. More importantly, the later isolated strains of VISA-DRSA and VISA-DRSA-LRSA phenotype appeared to be directly diverged from the Clade Ia strains in this critical period of time. The speculation was supported by the time-scaled phylogeny that the MRCA of the Clade II and III strains was closely related to a Clade Ia strain (LTF03) and predicted to appear during the fifth course of glycopeptide treatment (day 331.8, Figure 7). Two descendants of the MRCA appearing on day 393.7 and day 406.4 was, respectively, becoming the common ancestors of Clades Ia/III and Clades Ia/Ib/II. The glycopeptide monotherapy was not only ineffective in eliminating the bacteria but eventually driving the evolution of multi-resistance to other anti-MRSA agents through multiple genetic pathways. We believe that a more effective strategy with regimens that combine different classes of antimicrobial agents or early transition to an alternative treatment against MRSA will be required after the initial failed attempt to improve the outcome of patient with persistent bacteremia.

Clade II strains shared a MRCA with Clade Ib strains on day 662.5 though a majority of the strains were isolated on approximately day 1,200 of bacteremia. The Clade II strains were characterized with the colonies that are smaller, less hemolytic, and less pigmented (SCV phenotype) than the strain in other two clades and mostly exhibited resistance to linezolid in addition to vancomycin and daptomycin. These multiple phenotypic abnormalities suggest that the Clade II strains have alterations of global gene expression and/or cell signaling pathways. It has been demonstrated that the SCVs were heterogenous bacterial population and was usually unstable and dynamic during the chronic infections (Proctor et al., 2006; Garcia et al., 2013; Tuchscherr et al., 2016). Before identification of the first LRSA strains (LTF22), the patient had undergone eight courses of linezolid treatment for a total of 144 days during approximately 24 months of bacteremia. The LRSA phenotype was rarely identified in *S. aureus*, with an estimated rate of 0.05% for clinical isolates (Gu et al., 2013). Our observation was consistent with previous report that the patients were usually treated with linezolid prior to isolation of LRSA for a mean duration of 20 months (Gu et al., 2013). The multi-resistant strains appeared to be inhibited by administration of TMP-SXT, tigecycline, and glycopeptide in this study, but one VISA-DRSA-LRSA strain (LTF31) shared the MRCA with other Clade II strains on day 905.5 still escaped the intensive course of anti-MRSA treatment. The emergence of LRSA phenotype in MRSA strains remained a significant treatment challenge.

Clade III strains had 26.2- and 3.2-fold more mutations than that in the Clade I and Clade II strains, respectively. However, the colony morphology in Clade III strains was not significantly affected when compared to Clade I strains. A majority of the mutations in Clade III strains appeared not directly related to the drug resistance. Indeed, with a total of 390 SNPs or INDELs in Clade III strains, only 12 (3.1%) mutations was present in two VISA/DRSA strains but absent in the VSSA strain suggesting association with VISA/DRSA phenotype. The proportion of VISA/DRSA-associated mutations to all SNPs or INDELs was significantly greater for Clade II strains (51/225, 22.7%, Table 2). In addition to loss of all three plasmids, the Clade III strains were characterized with loss the beta-hemolysin prophage carrying the immune evasion cluster (IEC) including *sak*, *scn*, *chp*, and *sep* genes. Loss of the IEC in the multi-resistant strains suggested that the dispensability of the genes may confer adaptive advantage for MRSA in an environment with highly selective pressure.

We did have limitations in this study. First, the very first MRSA isolate in this case was not available and the evolution of the MRSA strains during the first 3 months of bacteremia were not captured in this study. However, the study was aimed to delineate the genomic evolution of MRSA during the transition from susceptible phenotypes to tripe-resistance to vancomycin, daptomycin, and linezolid. The first available isolate (LTF01) remained susceptible to the three antimicrobial agents, and the important genetic changes related to the incremental drug resistance should have been revealed in this study. Second, it has been reported that the evolutionary rate of bacteria might be negatively associated with the sampling time. The 6.4-fold faster clock rate for the MRSA strains in persistent bacteremia than that of the contemporary MRSA strains in the general health-care facilities might be offset to certain degree due to the shorter sampling time for the successive strains (approx. 3 years vs. 17 years).

## Conclusion

Genomic evolution of a MRSA ST5 strain during long-term persistent bacteremia on aggressive antimicrobial therapy was approximately 6.4-fold faster than that of the contemporary ST5 clinical isolates. The development of incremental resistance to vancomycin, daptomycin, and linezolid was coupled with substantial morphological changes and proceeded through multiple pathways. Both mutations and recombinations were common events during the evolution. While the mutations were frequently associated with the development of drug resistance, the recombination resulting in the gene loss appeared to provide adaptive advantage in harsh host environment rather than mediating drug non-susceptibility.

## Data Availability Statement

The datasets presented in this study can be found in online repositories. The names of the repository/repositories and accession number(s) can be found at: https://www.ncbi.nlm.nih.gov/genbank/, PRJNA495118.

## Author Contributions

C-JC and Y-CH designed the study. C-JC and S-SS performed the samples and data collection and conducted the genome sequencing and data analysis. C-JC wrote the manuscript. All authors contributed to the article and approved the submitted version.

## Conflict of Interest

The authors declare that the research was conducted in the absence of any commercial or financial relationships that could be construed as a potential conflict of interest.

## Footnotes

1 http://itol.embl.de

2 https://github.com/rambaut/figtree/releases

3 http://www.nmpdr.org/FIG/wiki/view.cgi/FIG/RapidAnnotationServer

4 http://github.com/samtools/bcftools

5 http://omgenomics.com/circa

6 http://www.mgc.ac.cn/VFs

## Supplementary Material

TABLE S1 MLST, *spa* types and SCC*mec* types in 32 serial MRSA isolates from a patient with persistent bacteremia.

TABLE S2 Information of 71 non-duplicated clinical MRSA strains belonging to ST5 or its single locus variants collected island wide from 1997 to 2014 in Taiwan.

TABLE S3 Non-synonymous SNPs and short INDELs strains across different clades, comparison to the parenteral strain, LTF01.

TABLE S4 The comparisons of numbers of SNPs/INDELs between the VISA strains and VSSA strains. The median and range are shown. Statistic was not performed for Clade II and Clade III strains due to small number of strains in the two clades: **(A)** comparisons of number of all SNPs/INDELs including synonymous and non-synonymous mutations; **(B)** only non-synonymous mutations were included for comparisons; and **(C)** only non-synonymous and clade-specific mutations were included for comparisons.

DATA SHEET 1 The positions of mutations, altered nucleotides, effect of the mutations, and the affected alleles among the successive MRSA isolates from a patient with persistent bacteremia. The first available isolates LTF01 was used as the comparator.

DATA SHEET 2 VISA/DRSA-associated genetic alteration among the successive MRSA strains. The positions of mutations, altered nucleotides, effect of the mutations, and function of the affected alleles are displayed.

PRESENTATION 1 The colony morphologies on blood agar and BM agar for 32 successive MRSA blood isolates from a patient with persistent bacteremia.
