# Supplementary material for: Evolution of Multi-Resistance to Vancomycin, Daptomycin, and Linezolid in Methicillin-Resistant Staphylococcus aureus Causing Persistent Bacteremia
Source: Front Microbiol. 2020 Jul 7;11:1414. doi: 10.3389/fmicb.2020.01414 (PMC7381330; doi:10.3389/fmicb.2020.01414)
Supplement: TABLE S1 — MLST, spa types and SCCmec types in 32 serial MRSA isolates from a patient with persistent bacteremia. [file Table_1.DOCX]

Supplementary file Table 1. MLST, *spa* types and SCC*mec* types in 32 serial MRSA isolates from a patient with persistent bacteremia

| Strain | Isolation date (y/m/d) | MLST | SCC*mec* | *spa* type |
| --- | --- | --- | --- | --- |
|  |  |  |  |  |
| LTF01 | 2006/9/8 | ST5 | II | t002 |
| LTF02 | 2006/10/27 | ST5 | II | t002 |
| LTF03 | 2006/11/20 | ST5* | II | t002 |
| LTF04 | 2007/3/4 | ST5* | II | t002 |
| LTF05 | 2007/4/3 | ST5* | II | t002 |
| LTF06 | 2007/4/8 | ST5* | II | t002 |
| LTF07 | 2007/4/19 | ST5* | II | t002 |
| LTF08 | 2007/7/6 | ST5 | II | t002 |
| LTF09 | 2007/7/20 | ST5 | II | t002 |
| LTF10 | 2007/8/19 | ST5* | II | t002 |
| LTF11 | 2007/11/7 | ST5* | II | t002 |
| LTF12 | 2008/2/13 | ST5* | II | t002 |
| LTF13 | 2008/5/7 | ST5* | II | t002 |
| LTF14 | 2008/6/4 | ST5* | II | t002 |
| LTF15 | 2008/8/3 | ST5* | II | t002 |
| LTF16 | 2008/8/16 | ST5* | II | t002 |
| LTF17 | 2008/12/3 | ST5* | II | t002 |
| LTF18 | 2008/12/12 | ST5* | II | t002 |
| LTF19 | 2009/2/25 | ST5* | II | t002 |
| LTF20 | 2009/3/18 | ST5* | II | t002 |
| LTF21 | 2009/4/14 | ST5* | II | t002 |
| LTF22 | 2009/4/20 | ST5* | II | t002 |
| LTF23 | 2009/4/24 | ST5* | II | t13754 |
| LTF24 | 2009/4/29 | ST5* | II | t002 |
| LTF25 | 2009/4/29 | ST5* | II | t002 |
| LTF26 | 2009/5/5 | ST5* | II | t002 |
| LTF27 | 2009/5/14 | ST5* | II | t002 |
| LTF28 | 2009/8/5 | ST5* | II | t002 |
| LTF29 | 2009/8/11 | ST5* | II | t002 |
| LTF30 | 2009/8/16 | ST5* | II | t002 |
| LTF31 | 2009/9/12 | ST5* | II | t002 |
| LTF32 | 2009/9/27 | ST5* | II | t002 |

* Single locus variants (SLVs) of ST5, with one nucleotide mismatch (T252A) in the *gmk* allele for all SLV strains.

Abbreviations: MLST, multi-locus sequence type; SCC*mec*, staphylococcal chromosomal cassette *mec*; *spa*, staphylococcal protein A
