# Supplementary material for: Evolution of Multi-Resistance to Vancomycin, Daptomycin, and Linezolid in Methicillin-Resistant Staphylococcus aureus Causing Persistent Bacteremia
Source: Front Microbiol. 2020 Jul 7;11:1414. doi: 10.3389/fmicb.2020.01414 (PMC7381330; doi:10.3389/fmicb.2020.01414)
Supplement: TABLE S2 — Information of 71 non-duplicated clinical MRSA strains belonging to ST5 or its single locus variants collected island wide from 1997 to 2014 in Taiwan. [file Table_2.DOCX]

Supplementary file Table 2. Information of 71 non-duplicated clinical MRSA strains belonging to ST5 or its single locus variants collected island-wide from 1997 to 2014 in Taiwan.

| Strain | Isolation Year | Source | MLST |
| --- | --- | --- | --- |
| 441 | 1997 | NA | ST5 |
| 1675 | 2004 | NA | ST5 |
| 4818 | 2001 | Blood | ST5 |
| 7826 | 2002 | NA | ST5 |
| 8749 | 2000 | NA | ST5 |
| 9180 | 2001 | NA | ST5 |
| GC-17 | 2000 | Catheter | ST5 |
| GC-2 | 2000 | Wound | ST5 |
| JGR1 | 2010 | Blood | ST5 |
| JGR6 | 2010 | Blood | ST5 |
| KTR42 | 2010 | Blood | ST5 |
| KTR50 | 2010 | Blood | ST5 |
| LCR72 | 2010 | Blood | ST5 |
| LCR73 | 2010 | Blood | ST5 |
| MKR10 | 2010 | Blood | ST5 |
| MKR15 | 2010 | Blood | ST5 |
| MRSA12 | 2009 | Blood | ST5 |
| MRSA7 | 2009 | Blood | SLV (tpi_495/2snp) of ST5 |
| MRSA9 | 2009 | Blood | ST5 |
| NCHR31 | 2010 | Blood | ST5 |
| NCHR32 | 2010 | Blood | ST5 |
| OLR5 | 2010 | Blood | ST5 |
| OLR6 | 2010 | Blood | ST5 |
| OLR8 | 2010 | Blood | ST5 |
| ORSA214 | 2004 | Sputum | ST5 |
| ORSA239 | 2005 | Sputum | ST5 |
| ORSA308 | 2007 | NA | ST5 |
| QR506 | 2012 | Sputum | ST5 |
| QR513 | 2012 | Wound | ST5 |
| QR518 | 2012 | Sputum | ST5 |
| QR536 | 2012 | Sputum | ST5 |
| QR537 | 2012 | Bronchoalveolar lavage | ST5 |
| R134 | 2005 | Blood | ST5 |
| R152 | 2004 | Blood | ST5 |
| R158 | 2005 | Blood | ST5 |
| R71 | 2005 | Blood | ST5 |
| Sau10 | 2000 | Blood | ST5 |
| Sau104 | 2012 | Blood | ST5 |
| Sau108 | 2014 | Blood | ST5 |
| Sau113 | 2014 | Joint fluid | ST5 |
| Sau118 | 2014 | Blood | ST5 |
| Sau12 | 2000 | Pus | ST5 |
| Sau121 | 2014 | Blood | ST5 |
| Sau13 | 2000 | Respiratory | SLV (arcC_503/1snp) of ST5 |
| Sau22 | 2002 | Catheter | ST5 |
| Sau27 | 2002 | Catheter | ST5 |
| Sau29 | 2002 | Respiratory | ST5 |
| Sau30 | 2002 | Respiratory | ST5 |
| Sau33 | 2004 | Respiratory | ST5 |
| Sau36 | 2004 | Blood | ST5 |
| Sau42 | 2004 | Catheter | ST5 |
| Sau46 | 2004 | Blood | ST5 |
| Sau47 | 2006 | Blood | ST5 |
| Sau50 | 2006 | Blood | ST5 |
| Sau60 | 2006 | Blood | ST5 |
| Sau63 | 2008 | Ear | ST5 |
| Sau65 | 2008 | Body fluid | ST5 |
| Sau72 | 2008 | Blood | ST5 |
| Sau74 | 2008 | Blood | ST5 |
| Sau78 | 2008 | Blood | ST5 |
| Sau79 | 2010 | Catheter | ST5 |
| Sau81 | 2010 | Blood | ST5 |
| Sau85 | 2010 | Catheter | ST5 |
| Sau87 | 2010 | Catheter | ST5 |
| Sau91 | 2010 | Blood | ST5 |
| Sau97 | 2012 | Blood | ST5 |
| Sau98 | 2012 | Blood | ST5 |
| Sau99 | 2012 | Blood | ST5 |
| Tsa-62 | 2001 | Pleural effusion | ST5 |
| Tsa-65 | 2001 | Ascites | ST5 |
| XR38 | 2015 | Wound | ST5 |

Abbreviations: ST, sequence type; SLV, single locus variant;
