# Supplementary material for: Evolution of Multi-Resistance to Vancomycin, Daptomycin, and Linezolid in Methicillin-Resistant Staphylococcus aureus Causing Persistent Bacteremia
Source: Front Microbiol. 2020 Jul 7;11:1414. doi: 10.3389/fmicb.2020.01414 (PMC7381330; doi:10.3389/fmicb.2020.01414)
Supplement: TABLE S3 — Non-synonymous SNPs and short INDELs strains across different clades, comparison to the parenteral strain, LTF01. [file Table_3.DOCX]

Supplementary file Table 3. Non-synonymous SNPs and short INDELs strains across different clades, comparison to the parenteral strain, LTF01

| Nucleotide position in N315 genome | Reference nucleotide(s) | Altered nucleotide(s) | Affected allele | Amino acid change | Clade Ia | | | | | | | | | | | | Clade Ib | | | | | | | | | Clade II | | | | | | | Clade III | | |
| --- | --- | --- | --- | --- | --- | --- | --- | --- | --- | --- | --- | --- | --- | --- | --- | --- | --- | --- | --- | --- | --- | --- | --- | --- | --- | --- | --- | --- | --- | --- | --- | --- | --- | --- | --- |
|  |  |  |  |  | LTF 2 | LTF 3 | LTF 4 | LTF 5 | LTF 6 | LTF 7 | LTF 8 | LTF 9 | LTF10 | LTF11 | LTF13 | LTF14 | LTF12 | LTF15 | LTF19 | LTF21 | LTF27 | LTF28 | LTF29 | LTF30 | LTF32 | LTF20 | LTF22 | LTF23 | LTF24 | LTF25 | LTF26 | LTF31 | LTF16 | LTF17 | LTF18 |
| 375569 | G | A | SA0317 | Glu326Lys |  |  |  |  |  |  |  |  | ■ | ■ | ■ | ■ |  |  |  |  |  |  |  |  |  |  |  |  |  |  |  |  | ■ | ■ | ■ |
| 581065 | A | C | rpoB | Lys482Asn |  |  |  |  | ■ |  | ■ | ■ | ■ | ■ | ■ | ■ |  |  |  |  |  |  |  |  |  |  |  |  |  |  |  |  | ■ | ■ | ■ |
| 581205 | C | T | rpoB | Ser529Leu |  |  |  |  |  |  |  |  |  |  |  |  | ■ | ■ | ■ | ■ | ■ | ■ | ■ | ■ | ■ | ■ | ■ | ■ | ■ | ■ | ■ | ■ |  |  |  |
| 880673 | G | T | SA0774 | Gly188Val |  |  |  |  |  |  |  |  | ■ | ■ | ■ | ■ |  |  |  |  |  |  |  |  |  |  |  |  |  |  |  |  | ■ | ■ | ■ |
| 1080441 | G | T | potD | Lys292Asn |  |  |  |  |  |  |  |  |  |  |  |  | ■ | ■ | ■ | ■ | ■ | ■ | ■ | ■ | ■ | ■ | ■ | ■ | ■ | ■ | ■ | ■ |  |  |  |
| 1172512 | A | T | ileS | Lys401Asn |  |  |  |  |  |  |  |  | ■ | ■ | ■ | ■ |  |  |  |  |  |  |  |  |  |  |  |  |  |  |  |  | ■ | ■ | ■ |
| 1423805 | T | A | truncated-arlR | Asn128Ile |  |  |  |  |  |  |  |  |  |  | ■ | ■ |  |  |  |  |  |  |  |  |  |  |  |  |  |  |  |  |  | ■ |  |
| 1469450 | G | A | ebhB | Thr118Ile |  |  |  |  |  |  |  |  |  |  |  |  | ■ | ■ | ■ | ■ | ■ | ■ | ■ | ■ | ■ | ■ | ■ | ■ | ■ | ■ | ■ | ■ |  |  |  |
| 1547787 | C | A | malR | Glu71* |  |  |  |  |  |  |  |  |  |  |  |  | ■ | ■ | ■ | ■ | ■ | ■ | ■ | ■ | ■ | ■ | ■ | ■ | ■ | ■ | ■ | ■ |  |  |  |
| 1547903 | G | A | malR | Thr32Ile |  |  |  |  |  |  |  |  | ■ | ■ | ■ | ■ |  |  |  |  |  |  |  |  |  |  |  |  |  |  |  |  | ■ | ■ | ■ |
| 1646261 | A | T | SA1445 | Ile64Asn |  |  |  |  |  |  |  |  | ■ | ■ | ■ | ■ |  |  |  |  |  |  |  |  |  |  |  |  |  |  |  |  | ■ | ■ | ■ |
| 1911408 | C | T | SA1674 | Val18Ile |  |  |  |  |  |  |  |  |  |  |  |  | ■ | ■ | ■ | ■ | ■ | ■ | ■ | ■ | ■ | ■ | ■ | ■ | ■ | ■ | ■ | ■ |  |  |  |
| 1947621 | A | T | vraS | Ser262Thr |  |  |  |  |  |  |  |  |  | ■ | ■ | ■ |  |  |  |  |  |  |  |  |  |  |  |  |  |  |  |  | ■ | ■ | ■ |
| 2005335 | A | G | truncated(hlb) | Arg63Gly |  |  |  |  |  |  |  |  | ■ | ■ | ■ | ■ |  |  |  |  |  | ■ | ■ |  |  |  |  |  |  |  |  |  | ■ | ■ | ■ |
| 2035244 | T | G | SA1788 | Asn11His | ■ |  |  |  |  |  |  |  | ■ | ■ | ■ | ■ |  |  |  |  | ■ | ■ | ■ | ■ |  |  | ■ |  |  |  |  |  | ■ | ■ |  |
| 2035249 | G | C | SA1788 | Thr9Arg |  |  |  |  |  |  |  |  | ■ | ■ | ■ | ■ |  |  |  |  |  | ■ | ■ | ■ |  |  | ■ |  |  |  |  |  | ■ | ■ |  |
| 2035264 | T | G | SA1788 | Lys4Thr |  |  |  |  |  |  |  |  | ■ | ■ | ■ | ■ |  |  |  |  |  | ■ | ■ |  |  |  |  |  |  |  |  |  | ■ | ■ |  |
| 2035267 | C | T | SA1788 | Arg3Lys | ■ |  |  |  |  |  |  |  | ■ | ■ | ■ | ■ |  |  |  |  |  | ■ | ■ |  |  |  |  |  |  |  |  |  | ■ | ■ |  |
| 2041411 | T | C | SA1797 | Thr5Ala |  |  |  |  |  |  | ■ | ■ | ■ | ■ | ■ | ■ | ■ |  |  |  | ■ | ■ | ■ | ■ | ■ |  |  |  |  |  |  |  | ■ | ■ |  |
| 2154196 | A | G | murA | Val25Ala |  |  |  |  |  |  |  |  |  | ■ | ■ | ■ |  |  |  |  |  |  |  |  |  |  |  |  |  |  |  |  | ■ | ■ | ■ |
