# Supplementary figures and images for: Evolution of Multi-Resistance to Vancomycin, Daptomycin, and Linezolid in Methicillin-Resistant Staphylococcus aureus Causing Persistent Bacteremia
Source: Front Microbiol. 2020 Jul 7;11:1414. doi: 10.3389/fmicb.2020.01414 (PMC7381330; doi:10.3389/fmicb.2020.01414)

Supplementary file Table 4

(A)


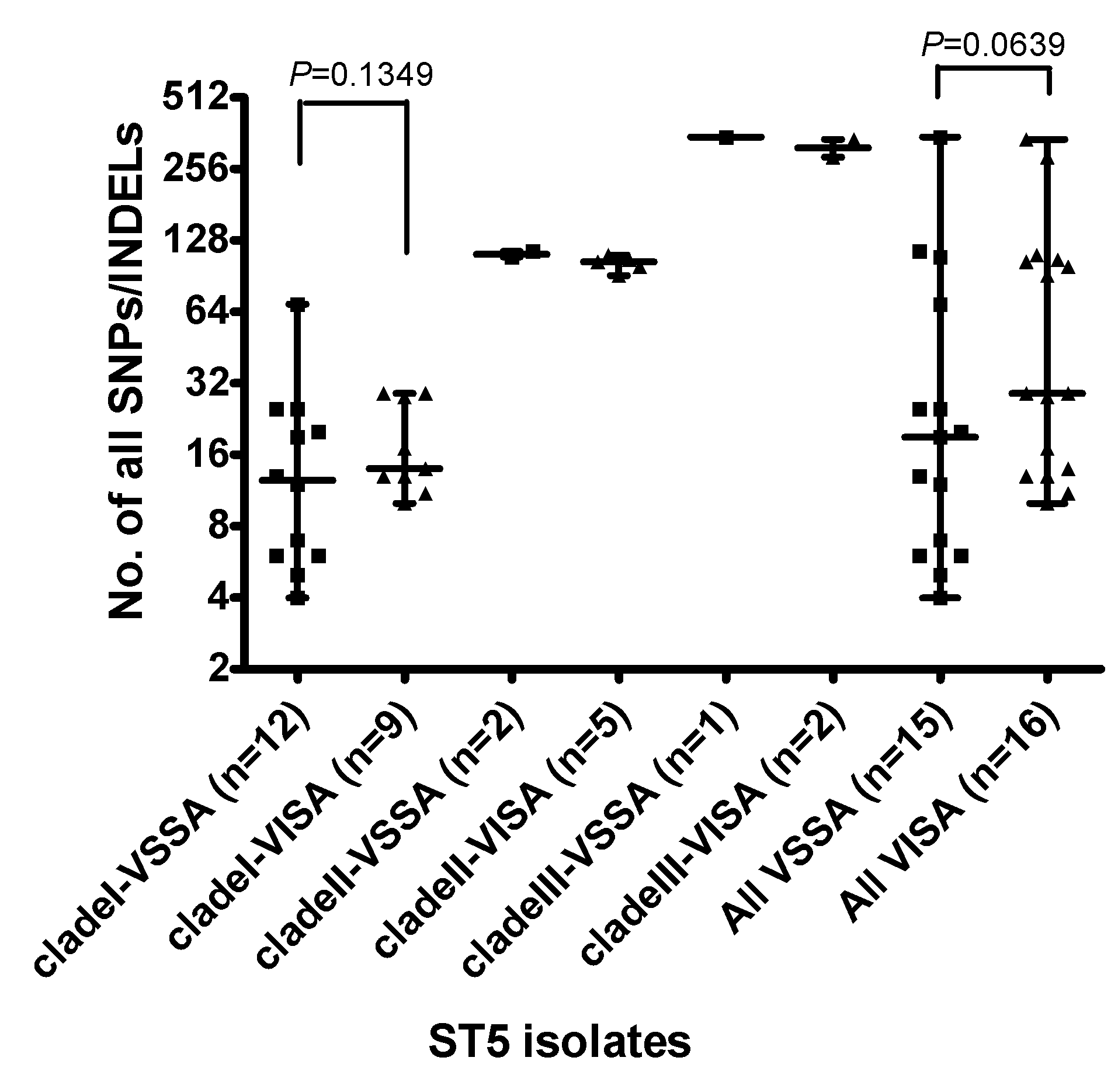


(B)


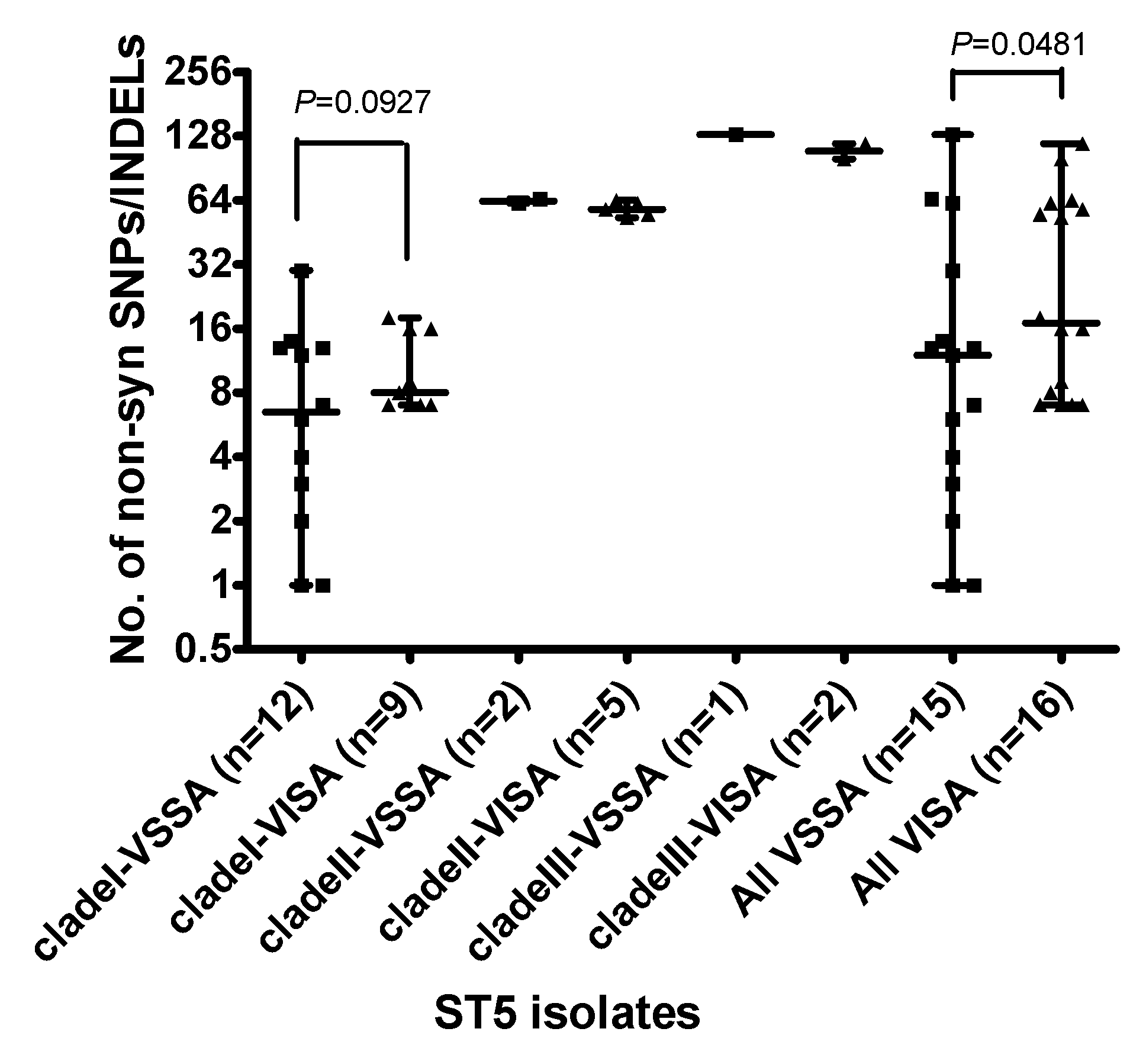


(C)


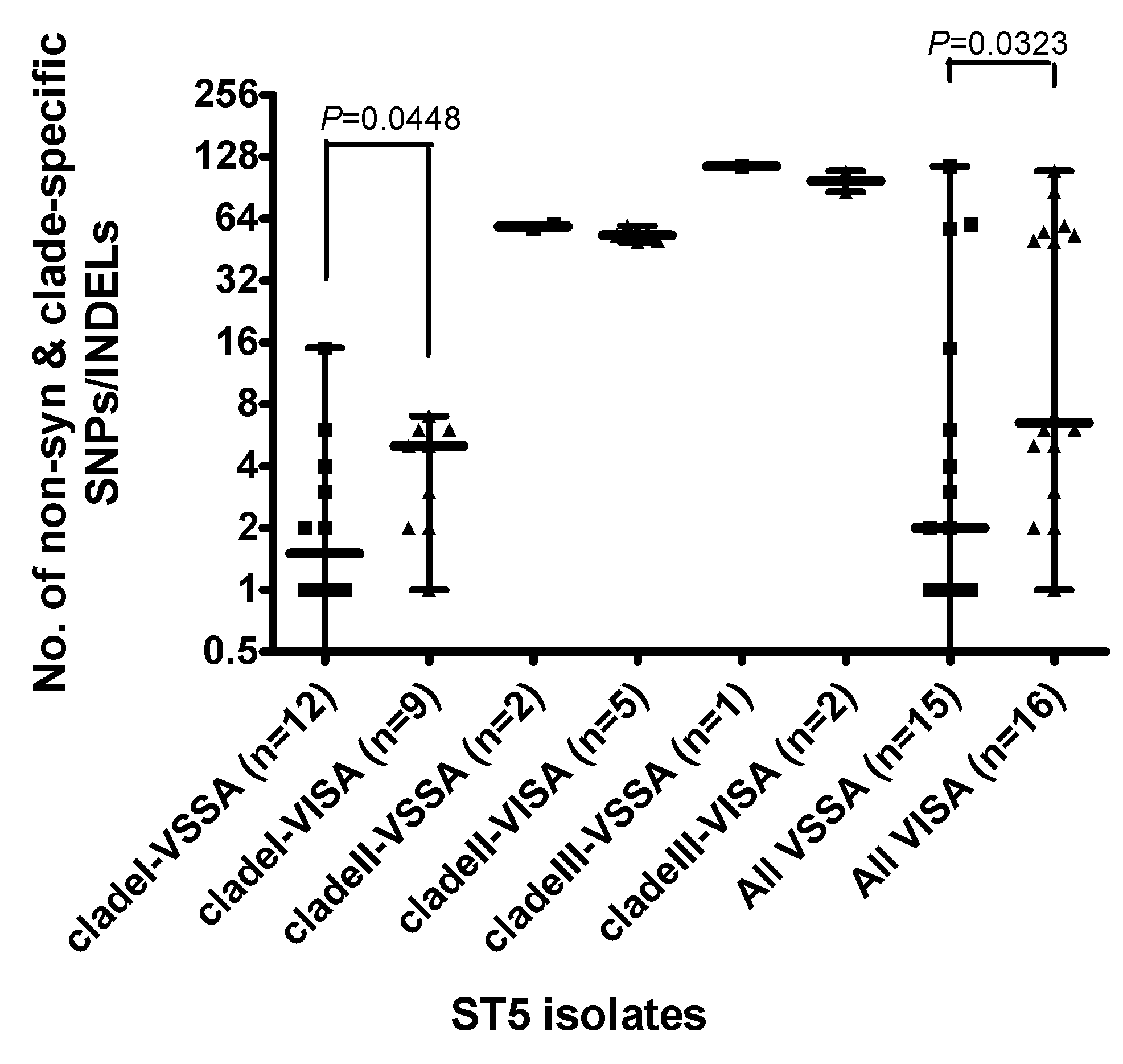

Supplement: TABLE S4 — The comparisons of numbers of SNPs/INDELs between the VISA strains and VSSA strains. The median and range are shown. Statistic was not performed for Clade II and Clade III strains due to small number of strains in the two clades: (A) comparisons of number of all SNPs/INDELs including synonymous and non-synonymous mutations; (B) only non-synonymous mutations were included for comparisons; and (C) only non-synonymous and clade-specific mutations were included for comparisons. [file Table_4.DOCX]
