# Supplementary material for: Evolution of Multi-Resistance to Vancomycin, Daptomycin, and Linezolid in Methicillin-Resistant Staphylococcus aureus Causing Persistent Bacteremia
Source: Front Microbiol. 2020 Jul 7;11:1414. doi: 10.3389/fmicb.2020.01414 (PMC7381330; doi:10.3389/fmicb.2020.01414)
Supplement: PRESENTATION 1 — The colony morphologies on blood agar and BM agar for 32 successive MRSA blood isolates from a patient with persistent bacteremia. [file Presentation_1.PPTX]

## Slide 1
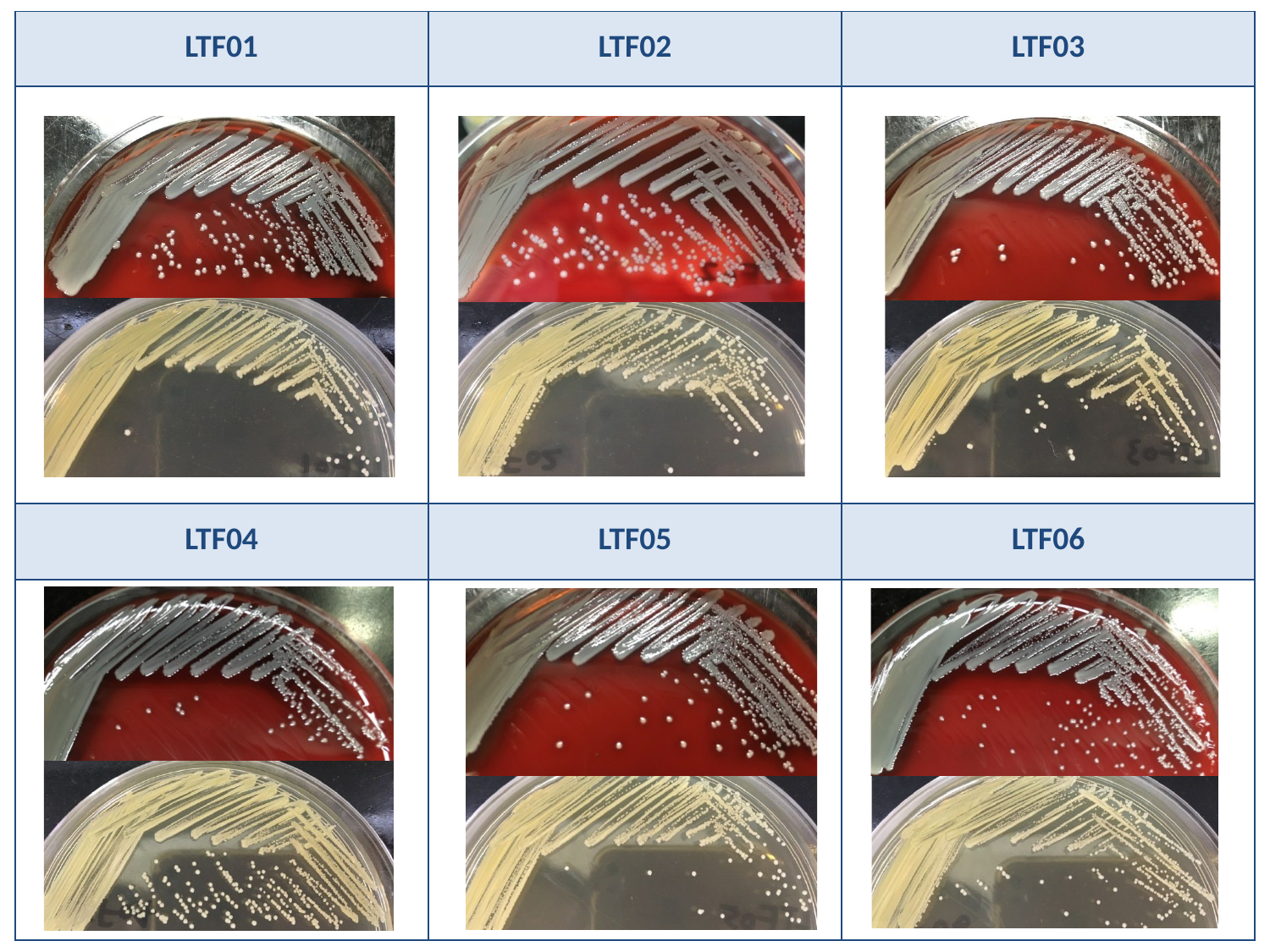

| LTF01 | LTF02 | LTF03 |
| --- | --- | --- |
| | | |
| LTF04 | LTF05 | LTF06 |
| | | |

## Slide 2
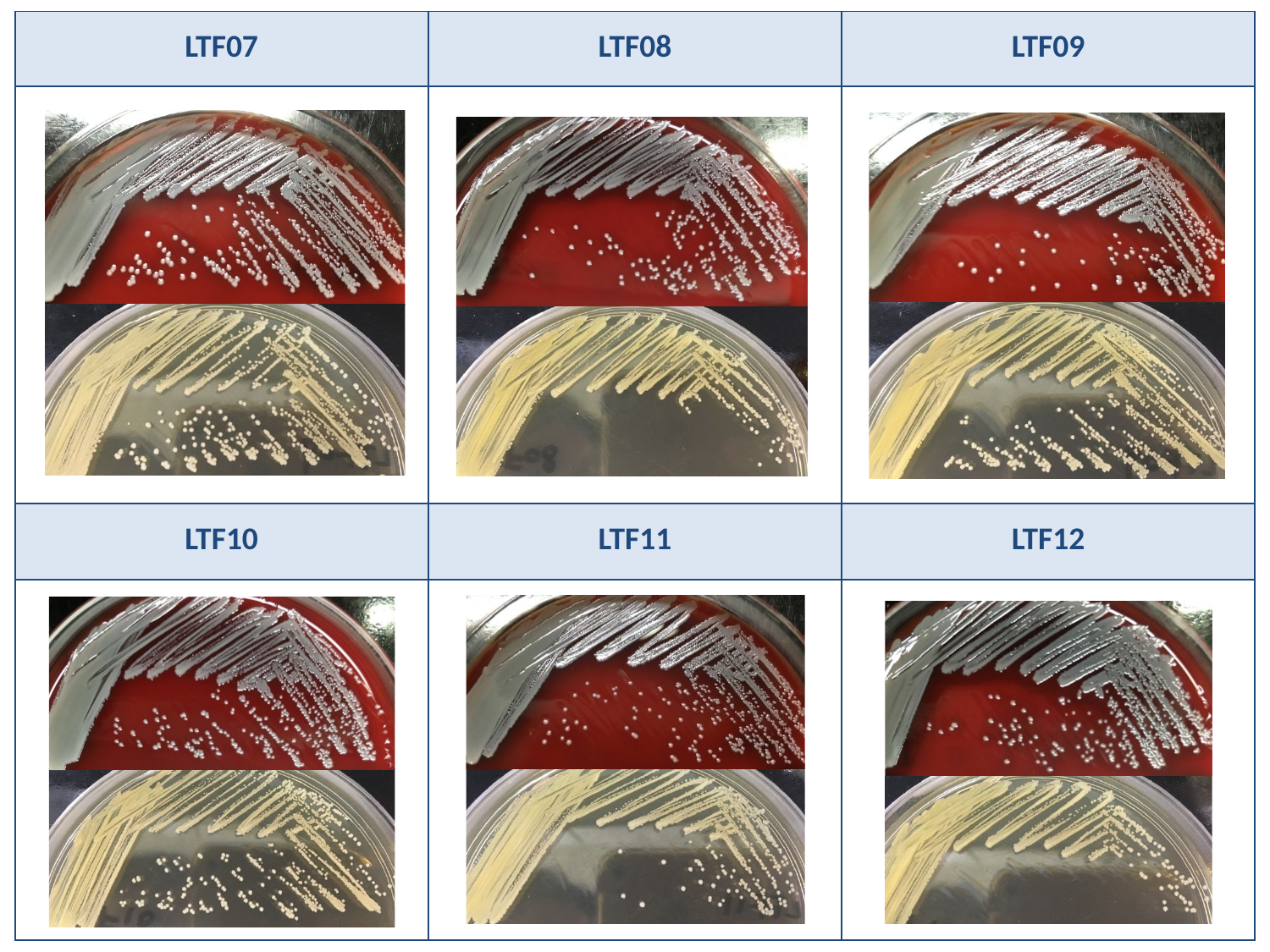

| LTF07 | LTF08 | LTF09 |
| --- | --- | --- |
| | | |
| LTF10 | LTF11 | LTF12 |
| | | |

## Slide 3
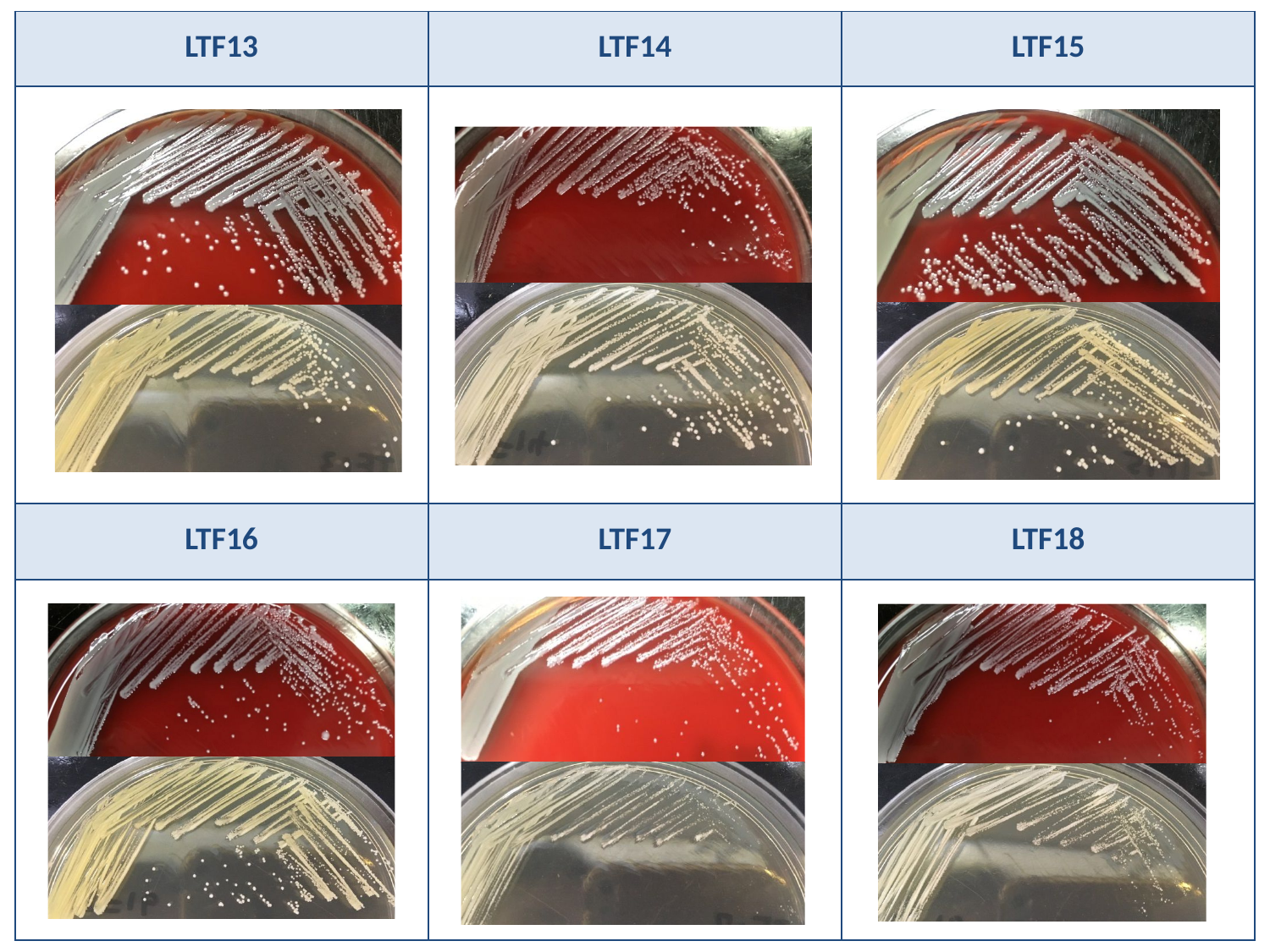

| LTF13 | LTF14 | LTF15 |
| --- | --- | --- |
| | | |
| LTF16 | LTF17 | LTF18 |
| | | |

## Slide 4
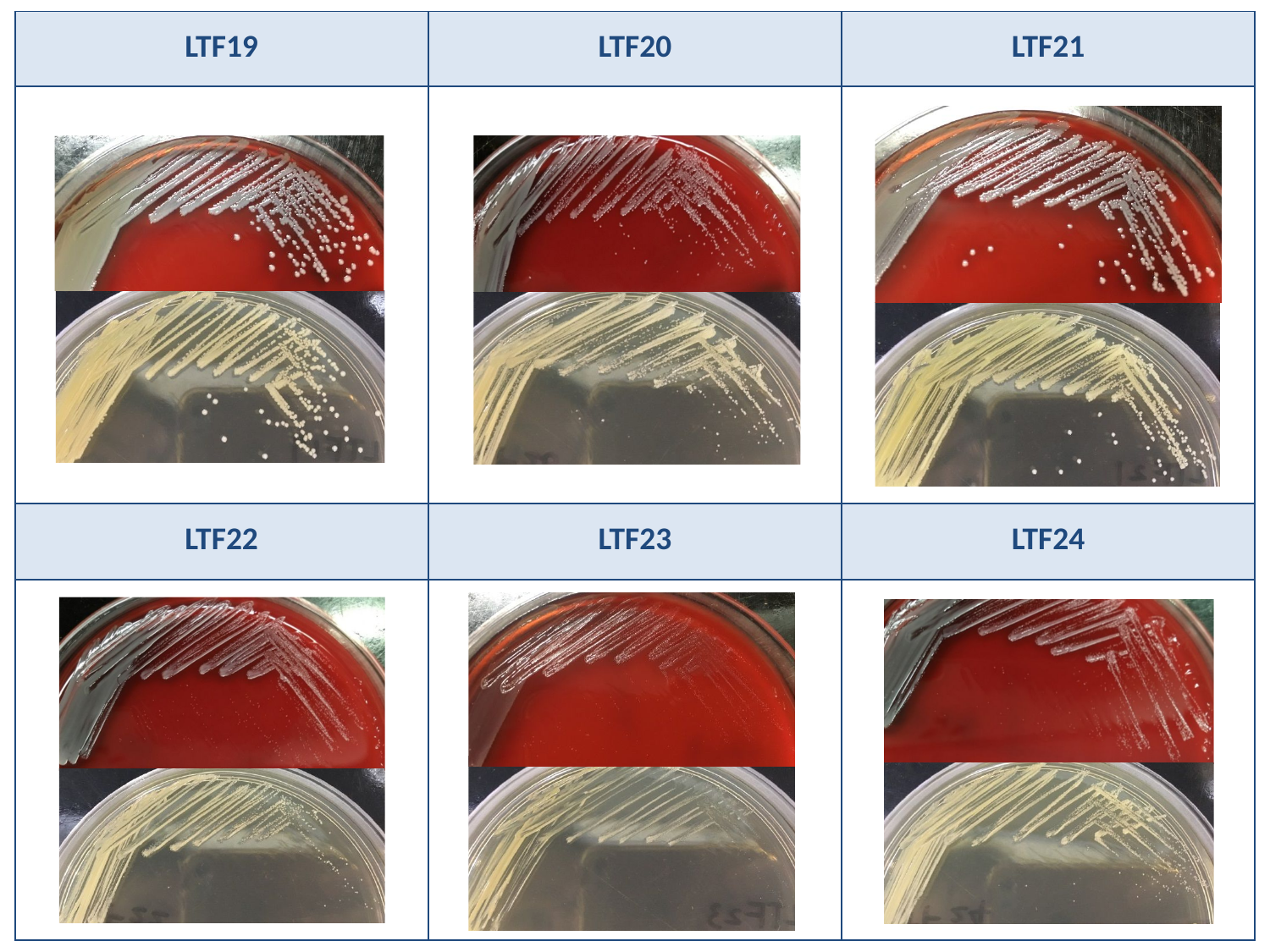

| LTF19 | LTF20 | LTF21 |
| --- | --- | --- |
| | | |
| LTF22 | LTF23 | LTF24 |
| | | |

## Slide 5
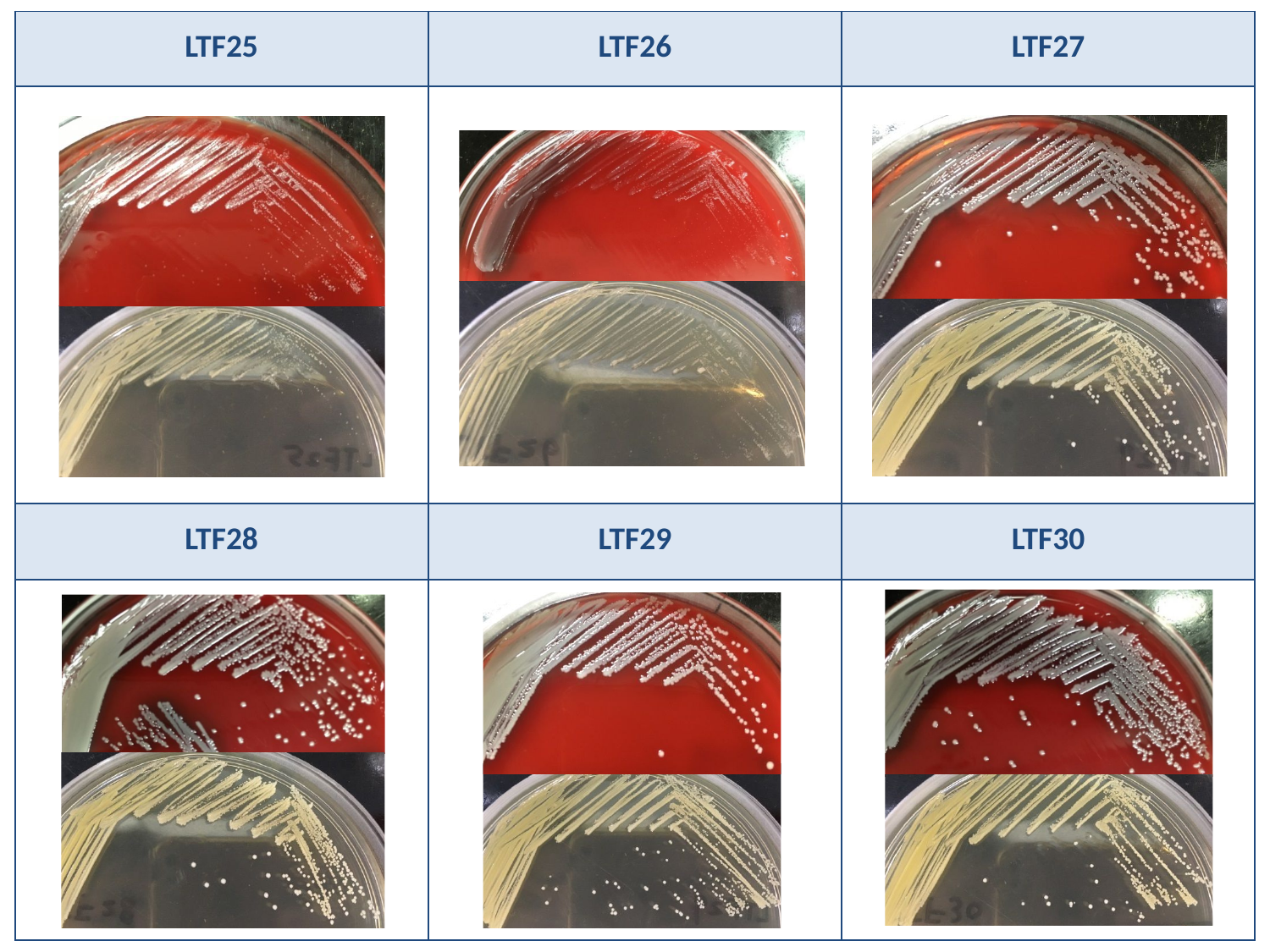

| LTF25 | LTF26 | LTF27 |
| --- | --- | --- |
| | | |
| LTF28 | LTF29 | LTF30 |
| | | |

## Slide 6
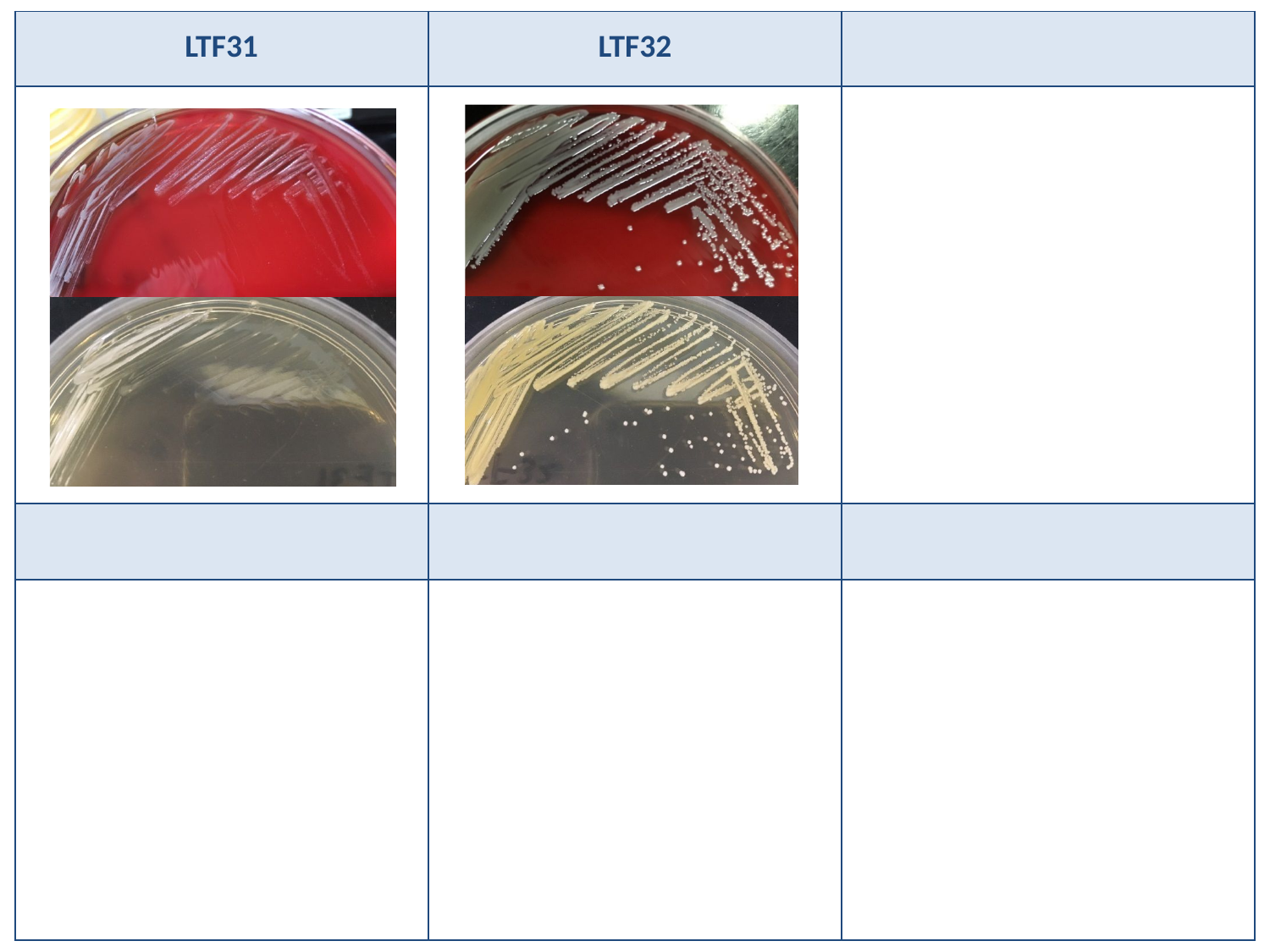

| LTF31 | LTF32 | |
| --- | --- | --- |
| | | |
| | | |
| | | |
